# Circular RNA Circ_0000098 Elevates ALX4 Expression *via* Adsorbing miR-1204 to Inhibit the Progression of Hepatocellular Carcinoma

**DOI:** 10.3389/fonc.2021.696078

**Published:** 2021-11-26

**Authors:** Ming Li, Wenjing Yue, Qiankun Li, Wenyu Yu, Yao Li, Xiaoling Cao

**Affiliations:** ^1^ Department of Gastroenterology, Yantai Affiliated Hospital of Binzhou Medical University, Yantai, China; ^2^ Medical Office, Yantai Affiliated Hospital of Binzhou Medical University, Yantai, China

**Keywords:** hepatocellular carcinoma, circ_0000098, miR-1204, ALX4, proliferation and metastasis

## Abstract

**Background:**

Circular RNAs (CircRNAs) feature prominently in the progression of various cancers. However, the biological functions of many circRNAs in hepatocellular carcinoma (HCC) are far from fully clarified. This work is performed to decipher the function of circ_0000098 (circSLC30A7) in modulating the progression of HCC and its molecular mechanism.

**Methods:**

Microarray data (GSE97332) were available from the Gene Expression Omnibus (GEO) database, and circRNA differentially expressed in HCC tissues was screened out by GEO2R tool. Circ_0000098, microRNA-1204 (miR-1204), and aristaless-like homeobox-4 (ALX4) mRNA expressions were detected by quantitative real-time polymerase chain reaction (qRT-PCR). Cell counting kit-8 (CCK-8), scratch wound healing, and Transwell assays were adopted to determine proliferation, migration, and invasion of HCC cells. ALX4 protein, E-cadherin, N-cadherin, and Vimentin expressions were evaluated by Western blot. In addition, the targeting relationship between miR-1204 and circ_0000098 or ALX4 was studied with dual-luciferase reporter assay and RIP assay.

**Results:**

Circ_0000098 expression level was markedly declined in HCC tissues and cells, and its underexpression was associated with larger tumor size of HCC patients. Knocking down circ_0000098 observably promoted the multiplication, migration, invasion, and epithelial-mesenchymal transition (EMT) of Huh7 and SMMC-7721 cells. Additionally, circ_0000098 was mainly distributed in the cytoplasm of HCC cells, and up-regulated ALX4 expression through competitively decoying miR-1204.

**Conclusion:**

Circ_0000098, as a competitive endogenous RNA (ceRNA) of miR-1204, upregulates ALX4 expression and suppresses the growth, migration, invasion, and EMT of HCC cells.

## Introduction

Liver cancer is one of the main causes of cancer-related death, with 905,677 new cases and 830,180 deaths worldwide in 2020 (1). Hepatocellular carcinoma (HCC) is the main pathological type of primary liver cancer, accounting for over 80% of primary liver cancer cases (2). In recent years, despite great advance in HCC treatment strategies, including surgical resection, liver transplantation, radiofrequency ablation, chemotherapy and so on, the prognosis of HCC patients is still far from satisfactory (3, 4). Therefore, it is pivotal to delve into the pathogenesis of HCC and find more effective therapeutics, to improve the survival time of HCC patients.

Circular RNAs (circRNAs) are non-coding RNA transcripts, which are produced by reverse splicing of precursor mRNA (5). CircRNAs have covalently closed structure without 5’ or 3’ ends, which are more stable than linear RNA and resistant to RNase R (6). Reportedly, circRNAs are vital in regulating multiple biological processes, such as cell growth, differentiation, apoptosis, and migration (7, 8). In addition, circRNAs, as reported, are abnormally expressed in various cancers and regulate cancer progression (9, 10). CircRNAs exerts their biological functions *via* different mechanisms. For instance, it can regulate the expression of the downstream genes by sponging microRNAs (miRNAs) (11, 12). For example, circ_0000092 up-regulates HN1 expression through competitively binding with miRNA-338-3p, thus promoting the progression of HCC (11). According to a previous report, miR-1204 expression level is increased in HCC, which can promote HCC cell viability and inhibit apoptosis by targeting ZNF418 to activate MAPK and c-Jun signaling (13). However, in HCC, whether miR-1204 is modulated by circRNA *via* competitive endogenous RNA (ceRNA) mechanism is still unclear.

In the present study, *via* bioinformatics analysis, circ_0000098 was screened out as a potential regulator in HCC. We report that circ_0000098 is markedly down-expressed in HCC tissues and cells, and it negatively regulates multiplication, migration, invasion, and epithelial-mesenchymal transition (EMT) of HCC cells. Besides, circ_0000098 regulates the expression of aristaless-like homeobox 4 (ALX4) by adsorbing miR-1204.

## Materials and Methods

### Bioinformatics Analysis

A dataset GSE97332 of circRNAs from 7 pairs of human HCC tumor tissue and normal hepar tissue was obtained from the Gene Expression Omnibus (GEO) database (https://www.ncbi.nlm.nih.gov/geo/). And the expression profile of circRNAs in normal hepar tissue and HCC tissue was analyzed by GEO2R. *P* < 0.05 and │log2 (Fold Change)│ > 2 were used as the threshold for screening out the circRNAs with differential expressions. Volcano plots were used to show the upregulated and downregulated circRNAs in the dataset. Heat maps were used to show the 10 circRNAs that were significantly up-regulated and the 10 circRNAs that were significantly down-regulated in HCC tissues.

### Clinical Specimens

34 pairs of HCC tissues and matched adjacent tissues were collected from HCC patients receiving surgery in the Hospital. Immediately after surgical resection, tissue samples were stored in liquid nitrogen until further analysis. None of the patients receive chemotherapy or radiotherapy before the operation. This research, with signed informed consent from the patients, was endorsed by the Ethics Committee of the Hospital. The experiments concerning human tissues were conducted in line with the Declaration of Helsinki.

### Cell Culture

Human HCC cell lines (HepG2, SNU423, and SNU475) were obtained from American Type Culture Collection (ATCC, Rockville, MD, USA). A normal hepatocyte line (HL-7702) and human HCC cell lines (Huh-7 and SMMC-7721) were purchased from the Type Culture Collection of Chinese Academy of Sciences (Shanghai, China). All of the cells were cultured in Dulbecco’s modified Eagle’s medium (DMEM, Sigma, St. Louis, MO, USA) with 10% fetal bovine serum (FBS, Invitrogen, Carlsbad, CA, USA) at 37°C and in 5% CO_2_.

### Quantitative Real-Time Polymerase Chain Reaction (qRT-PCR)

The total RNA was extracted from tissues and cells by a TRIzol kit (Invitrogen, Carlsbad, CA, USA) and then reversely transcribed into cDNA by a Primescript™ RT kit (TaKaRa, Dalian, China) and random primers or oligo (dt)_18_ primers. A Mir-X™miRNA First-Strand Synthesis kit (Clontech, Shanghai, China) was used for the reverse transcription of miRNA. Then, qRT-PCR was performed on ABI 7300 rapid real-time PCR system (Applied Biosystems, Foster City, CA, USA) with a SYBR^®^Premix Ex TaqTM II kit (Takara, Dalian, China) with U6 and GAPDH as the internal references. The relative expression was calculated by the 2^-ΔΔCt^ method. Primer sequences are detailed in **Table 1**. In RNase R treatment assay, the total RNA (2 μg) was incubated at 37°C for 20 min with or without 3 U/μg RNase R. Then circ_0000098 expression was detected by qRT-PCR, respectively, with GAPDH as the control. For determining the subcellular localization of circ_0000098, A PARIS™ kit (Thermofisher Scientific, Waltham, MA, USA) was used for subcellular fractionation. Then Circ_0000098 expression in cytoplasm and nucleus of the cells was examined by qRT-PCR, with GAPDH and U6 as the cytoplasmic control and nuclear control, respectively.

**Table 1:** Primers used for qRT-PCR.

	Forward	Reverse
circ_0000098	5’-GGTGTAATTGCTTCTGCCATC-3’	5’-TAACAGAAGCTGCCAGTCCA-3’
ALX4	5’-ACACATGGGCAGCCTGTTTG-3’	5’-TGCTTGAGGTCTTGCGGTCT-3’
GAPDH	5’-ACAACTTTGGTATCGTGGAAGG-3’	5’-GCCATCACGCCACAGTTTC-3’
miR-1204	5’-CGTGGCCTGGTCTCCATTAT-3’	5’-GGAACGATACAGAGAAGATTAGC-3’
U6	5’-CTCGCTTCGGCAGCACA-3’	5’-AACGCTTCACGAATTTGCGT-3’
SLC30A7	5’-TTGCCATAGCCATGAAGTGA-3’	5’-GTCTGCTGGGTCCTGTTGTT-3’

### Actinomycin D Assay

Huh7 and SMMC-7721 cells were treated with 2 μg/mL actinomycin D (Amyjet, Wuhan, China) for 0, 4, 8, 12, and 24 h. After the cells were harvested, the expression levels of circ_0000098 and SLC30A7 mRNA were detected by qRT-PCR to verify the stability of circ_0000098.

### Cell Transfection

Empty plasmid vector (Vector), circ_0000098 overexpression plasmid (circ_0000098-OE), siRNA negative control (si-NC), siRNAs targeting circ_0000098 (si-circ_0000098#1 and si-circ_0000098#2), miRNA mimics control (miR-NC), miR-1204 mimics, miR-1204 inhibitors (miR-1204-in), negative controls (miR-in), siRNAs targeting ALX4 (si-ALX4), and its negative control (si-NC) were available from GenePharma Co., Ltd. (Shanghai, China). The cells were transfected with Lipofectamine™ 3000 (Invitrogen, Carlsbad, CA, USA). After 48 h, the transfection efficiency was detected by qRT-PCR.

### Cell Proliferation Assay

Cell proliferation was detected by a cell counting kit-8(CCK-8) kit (Beyotime, Jiangsu, China). To be specific, Huh7 and SMMC-7721 cells were transferred in a 96-well plate at a density of 2×10^3^ cells/well. At different time points (0, 1, 2, 3, 4, and 5 d), 100 µL of serum-free medium and 10 µL of CCK-8 solution were added to each well, followed by the incubation at 37°C for 1 h. Next, a ELX-800 spectrometer microplate reader (Bio-Tek Instruments, Winooski, VT, USA) was used to measure the absorbance at the wavelength of 450 nm.

### Wound Healing Assay

The cells were inoculated in a 6-well plate at a density of 1×10^5^ cells/well. When cells reached 80%~90% confluence, a sterile pipette was adopted to make a cell-free scratch. The the cells were cultured with serum-free medium. Wound healing was monitored at 0 and 24 h by an inverted optical microscope (Nikon, Tokyo, Japan).

### Transwell Assay

Cell migration and invasion were evaluated with Transwell chamber (pore size 8.0μm; Millipore, Billerica, MA, USA). The transfected Huh7 and SMMC-7721 cells (4×10^5^ cells) were resuspended in 200 µL of serum-free DMEM and then added into the upper chamber of Transwell chamber, and the bottom chamber was added with 500 μL of complete medium containing 10% FBS. After 24 h, the cells remaining on the upper membrane surface were removed. Moreover, the cells on the bottom surface were fixed in 4% paraformaldehyde and stained with 0.1% crystal violet (Beyotime, Jiangsu, China). Images were captured by an inverted optical microscope, and the number of cells in five random fields of view was counted. Matrigel (diluted 1: 9, Corning Incorporated, Corning, NY, USA) was used to cover the membrane in invasion assay, but it was not used during the process of migration assay.

### Dual-Luciferase Reporter Assay

The wild type (WT) or mutant type (MUT) of circ_0000098 or ALX4 3’-UTR sequence was amplified and cloned into the psiCHECK-2 vector (Promega, Madison, WI, USA) to construct luciferase reporter plasmid (circ_0000098-WT/MUT or ALX4 3’-UTR-WT/MUT). The reporter plasmids, together with miR-1204 mimic or miR-NC, were co-transfected into Huh7 and SMMC-7721 cells by Lipofectamine™ 3000. After 48 h, the luciferase activity was detected by the dual-luciferase reporter analysis kit (Promega, Madison, WI, USA).

### RNA Immunoprecipitation (RIP) Assay

RIP assay was conducted with a Magna RIP RNA binding protein immunoprecipitation kit (Millipore, Billerica, MA, USA). Huh7 and SMMC-7721 cells were lyzed by RIP lysis buffer, and 100 μL of whole-cell extract was incubated with RIPA buffer (Beyotime, Shanghai, China) containing magnetic beads coupled with human anti-Argonaute2 (Ago2) antibody (Millipore, Billerica, MA, USA) at 4°C 8 h. Normal mouse IgG (Millipore, Billerica, MA, USA) was employed as a negative control. The samples were rinsed with washing buffer and then incubated with proteinase K at 55°C for 30 min to separate RNA- protein complexes from magnetic beads. Ultimately, the immunoprecipitated RNA was extracted and analyzed by qRT-PCR.

### Western Blot Assay

The total proteins were extracted by RIPA lysis buffer (Beyotime, Shanghai, China), with the protein concentration determined by a BCA protein assay kit (Thermo Fisher Scientific, Rockford, IL, USA). The equal amount of proteins (30 μg/lane) were separated by SDS-PAGE and then transferred to PVDF membrane (Millipore, Billerica, MA, USA). After being blocked with 5% skimmed milk at ambient temperature for 1 h, the membrane was incubated overnight at 4°C with primary antibodies including anti-ALX4 (SC-33643, 1: 1000, Santa Cruz Biotechnology, Shanghai, China), anti-E-cadherin (ab76055, 1:1000, Abcam, Shanghai, China), anti-N-cadherin (ab280375, 1:1000, Abcam, Shanghai, China), anti-Vimentin (ab16700, 1:1000, Abcam, Shanghai, China), and anti-GAPDH (ab9484, 1: 1000, Abcam, Shanghai, China). After the membranes were washed by TBST, the membranes were incubated with horseradish peroxidase (HRP)-coupled secondary antibody (ab205719, 1: 2000, Abcam, Shanghai, China) for 0.5 h at room temperature. Ultimately, Clarity Max™Western ECL substrate (Bio-Rad, Hercules, CA, USA) was adopted to develop the protein bands.

### Lung Metastasis Model in Nude Mice

The *in vivo* study were approved by the Animal Care and Use Committee of Yantai Affiliated Hospital of Binzhou Medical University. BALB/c nude mice (6 weeks old, male) were used to establish the lung metastasis model. 1×10^6^ Huh7 cells with circ_0000098 depletion or the control cells were injected into the tail vein of each mouse (10 mice per group). After 3 weeks, the mice were killed, and lung tissues were harvested. Subsequently, the lung tissues were fixed, embedded in paraffin and sliced, and hematoxylin and eosin (H&E) staining was performed to detect the metastatic nodules.

### Statistical Analysis

All experiments were performed in triplicate. SPSS version 22.0 software (SPSS Inc., Chicago, IL, USA) was adopted for statistical analysis, with the data recorded as mean ± standard deviation (SD). Fisher’s exact test was employed to analyze the relationship between circ_0000098 expression and clinicopathological features. Student’s *t*-test was used to analyze the difference between two groups. One-way ANOVA was used to analyze the differences among the multiple groups. Pearson correlation analysis was employed to study the correlation among circRNA, miRNA, and mRNA expressions. *P*<0.05 denoted that the difference was of statistical significance.

## Results

### Circ_0000098 Was Lowly Expressed in HCC

**Figure 1:**
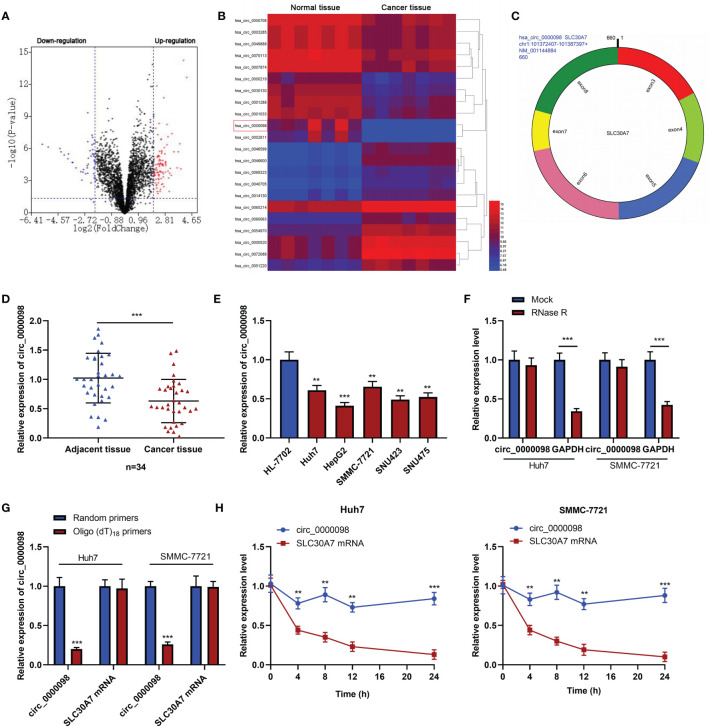
The expression of circ_0000098 was significantly down-regulated in HCC tissues and cells. **(A)** Volcano plot was used to show the expression changes of circRNAs in HCC tissues and normal tissues adjacent to cancer. CircRNAs whose expressions were significantly increased were marked in red, and those whose expressions were significantly decreased were marked in blue. **(B)** Heatmap showed differentially expressed circRNAs in HCC tissues relative to adjacent normal tissues. Red and blue colors indicate high-expression and low-expression, respectively. **(C)** The schematic diagram of circ_0000098 formation from SLC30A7 exons. **(D)** The expression of circ_0000098 in HCC tissues and adjacent tissues was detected by qRT-PCR. **(E)** qRT-PCR was used to detect the expression of circ_0000098 in HCC cells and HL-7702 cells. **(F)** Circ_0000098 and GAPDH mRNA expressions were detected by qRT-PCR after RNase R treatment, so as to determine the circular characteristics of circ_0000098. **(G)** The expressions of circ_0000098 and linear SLC30A7 mRNA were detected by qRT-PCR after total RNA was reversely transcribed using random primers or oligo (dt)_18_ primers. **(H)** The expressions of circ_0000098 and liner SLC30A7 mRNA were detected in Huh7 and SMMC-7721 cells by qRT-PCR after treatment with actinomycin **(D)** **P < 0.01 and ***P < 0.001.

GEO database (GSE97332) was adopted to identify differentially expressed circRNAs in HCC tissues compared with normal tissues adjacent to HCC. According to the criteria (log_2_|fold change |>2, *P*<0.05), there were 149 differentially expressed circRNAs in HCC tissues, of which 98 circRNAs were up-regulated and 51 circRNAs were down-regulated (**Figure 1A**). Circ_0000098 expression in HCC tissue was lower than that in normal tissues (**Figure 1B**). Circ_0000098 was derived from exons 3, 4, 5, 6, 7, and 8 of SLC30A7 gene (**Figure 1C**) (14). qRT-PCR indicated that circ_0000098 expression was markedly lower in HCC tissues than that in adjacent tissues (**Figure 1D**). Also, circ_0000098 expression in HCC cell lines, compared with that in HL-7702 cells, was significantly down-regulated (**Figure 1E**). To verify the circular structure of circ_0000098, we carried out RNase R treatment experiments in Huh7 and SMMC-7721 cells and found that circ_0000098 was resistant to RNase R while the expression of GAPDH mRNA was decreased significantly after RNase R treatment (**Figure 1F**). Furthermore, random primers or oligo (dT)_18_ primers were used to perform reverse transcription with total RNAs extracted from Huh7 and SMMC-7721 cells. The results showed that when oligo (dT)_18_ primers were used, the relative expression of circ_0000098 was significantly lower than when random primers were used, while the expression of linear SLC30A7 mRNA did not change significantly (**Figure 1G**). This indicated that circ_0000098 did not contain 3’ polyadenylated tail. In addition, actinomycin D assay demonstrated that circ_0000098 had a longer half-life time in Huh7 and SMMC-7721 cells compared with linear SLC30A7 mRNA (**Figure 1H**). This indicated that circ_0000098 was more stable than linear SLC30A7 mRNA. Besides, the Fisher’s exact test showed that the low expression of circ_0000098 was associated with the larger tumor size of HCC patients (**Table 2**). The results suggested that circ_0000098 might be related to the progression of HCC.

**Table 2:** Correlation between circ_0000098 expression and clinicopathological features in 34 HCC patients.

Characteristics	Number	circ_0000098 expression		*P* value
		Low	High	
Age(years)				
≤50	13	7	6	1.000
>50	21	10	11	
Gender				
Male	25	11	14	0.437
Female	9	6	3	
TMN stage				
I/II	10	2	8	0.059
III/IV	24	15	9	
Tumor size (cm)				
< 5	13	2	11	0.005*
≥ 5	21	15	6	
Vascular invasion				
Yes	20	11	9	0.727
No	14	6	8	
AFP (μg/L)				
≤400	12	5	7	0.720
>400	22	12	10	
Aetiology				
N.A.	9	4	5	0.839
Hepatitis B	15	7	8	
Alcoholic liver disease	6	4	2	
Fatty liver disease	4	2	2	

P value was calculated by Fisher’s exact test. *denotes p values less than 0.05.

### Circ_0000098 Inhibited the Proliferation, Migration, Invasion, and EMT of HCC Cells

**Figure 2:**
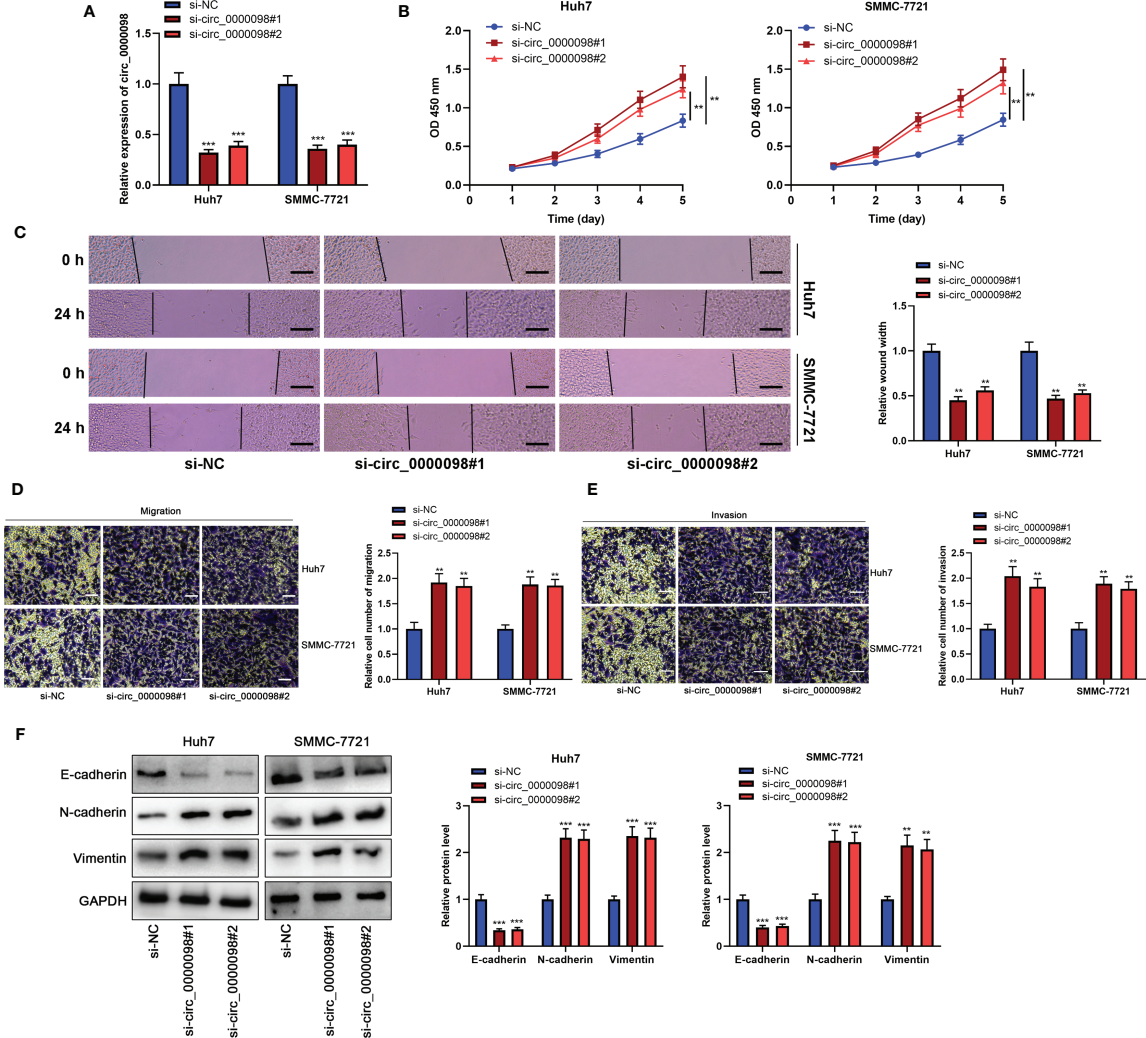
Knocking down circ_0000098 promoted the proliferation, migration, and invasion of HCC cells. **(A)** qRT-PCR was used to detect the expression of circ_0000098 in Huh7 and SMMC-7721 cells after the transfection of si-circ_0000098#1 and #2. **(B)** CCK-8 assay was used to evaluate cell proliferation. **(C)** The migration ability of cells was detected by wound healing assay (Scale bar, 100 μm). **(D, E)** Transwell assay was used to detect cell migration and invasion (Scale bar, 250 μm). **(F)** Western blot assay was used to detect the expression levels of E-cadherin, N-cadherin, and Vimentin in Huh7 and SMMC-7721 cells with down-regulated circ_0000098 expression. **P < 0.01 and ***P < 0.001.

To pinpoint the biological function of circ_0000098 in HCC, we transfected Huh7 and SMMC-7721 cells with two siRNAs targeting circ_0000098 to construct cell models of circ_0000098 knockdown (**Figure 2A**). CCK-8, wound healing, and Transwell assays showed that knocking down circ_0000098 significantly promoted the proliferation, migration, and invasion of Huh7 and SMMC-7721 cells (**Figures 2B**–**E**). Western blot assay verified that knockdown of circ_0000098 decreased the expression level of E-cadherin and up-regulated the expression levels of N-cadherin and Vimentin (**Figure 2F**). On the other hand, we transiently transfected the circ_0000098 overexpression plasmid into Huh7 and SMMC-7721 cells. Overexpression of circ_0000098 significantly inhibited proliferation, migration, invasion, and EMT of Huh7 and SMMC-7721 cells (**Supplementary Figures 1A**–**F**). These data implied that circ_0000098 modulated the malignant biological behaviors of HCC cells *in vitro*.

### Circ_0000098 Sponged miR-1204

**Figure 3:**
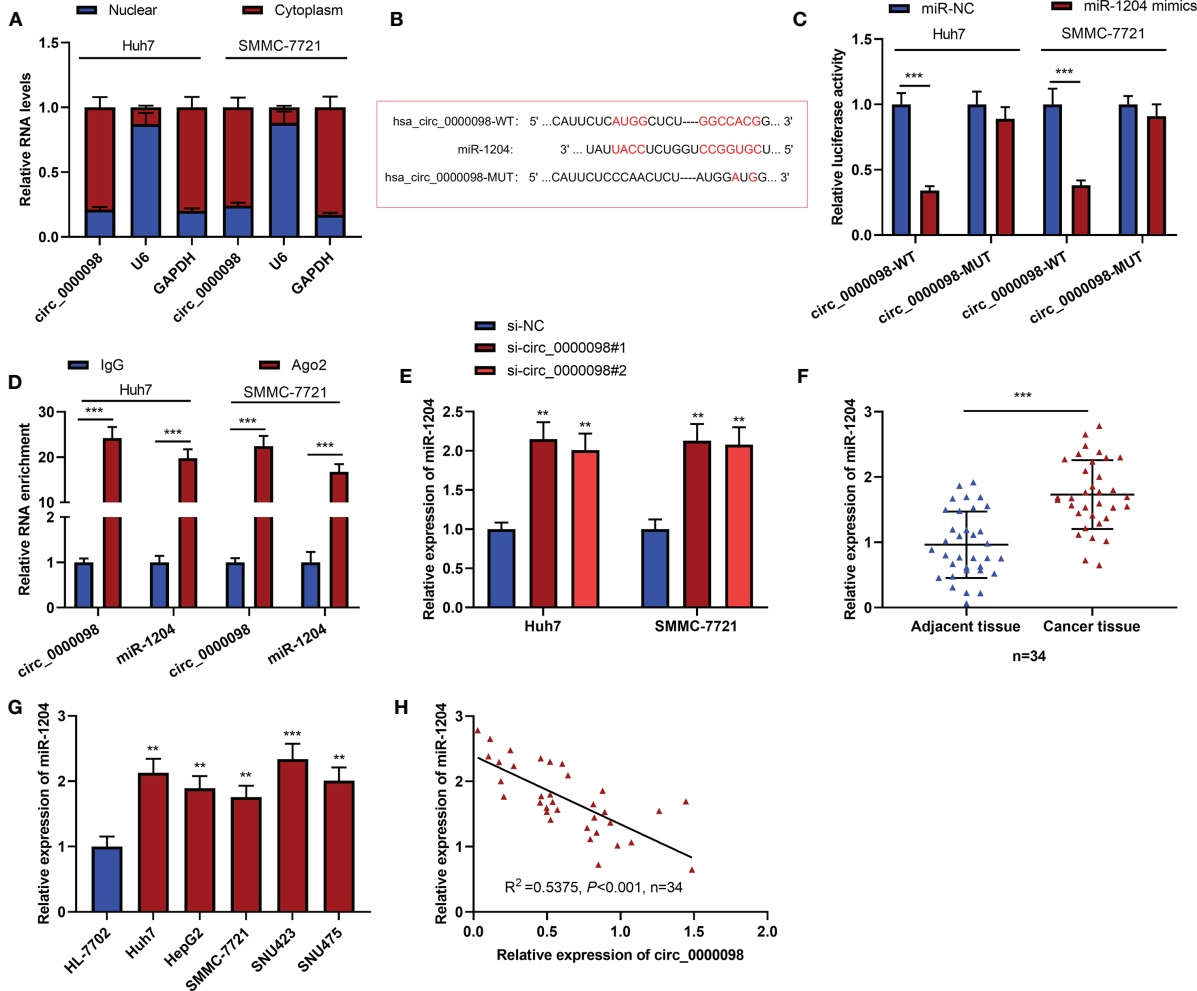
MiR-1204 was the target of circ_0000098. **(A)** qRT-PCR was used to detect the expressions of circ_0000098, U6, and GAPDH mRNA in the nucleus and cytoplasm of HCC cells. **(B)** The binding sequence between miR-1204 and circ_0000098 was predicted by Circular RNA Interactome database. **(C)** Dual-luciferase reporter assay was used to verify the binding relationship between miR-1204 and circ_0000098. **(D)** The direct interaction between circ_0000098 and miR-1204 was determined by RIP assay. **(E)** qRT-PCR was used to detect the expression of miR-1204 in cells transfected with si-circ_0000098#1 and #2. **(F, G)** The expression of miR-1204 in HCC tissues and cells was detected by qRT-PCR. **(H)** Pearson’s correlation analysis was used to analyze the correlation between miR-1204 and circ_0000098 expressions in 34 HCC tissues. **P < 0.01 and ***P < 0.001.

CircRNAs feature prominently in the cancer progression by adsorbing miRNAs (15). To reveal the hidden mechanism of circ_0000098 in regulating the HCC progression, we first analyzed the subcellular distribution of circ_0000098 and observed that circ_0000098 was predominantly located in the cytoplasm of HCC cells (**Figure 3A**). By searching the Circular RNA Interactome database (https://circinteractome.irp.nia.nih.gov) (16), we found putative binding sites between circ_0000098 and miR-1204, miR-1248, miR-1276, miR-136, miR-140-3p, miR-155, miR-183, miR-203, miR-337-3p, miR-346, miR-369-5p, etc. We mainly focused on exploring the role of miR-1204 (**Figure 3B**). Interestingly, high expression of miR-1204 expression was associated with the vascular invasion of the enrolled HCC patients (**Table 3**). Dual-luciferase reporter assay indicated that as against the control group, the up-regulation of miR-1204 expression markedly decreased the luciferase activity of circ_0000098-WT reporter, but no significant change was observed on the luciferase activity of circ_0000098-MUT reporter (**Figure 3C**). In addition, RIP analysis showed that circ_0000098 and miR-1204 were markedly enriched in Ago2 group as against the IgG group (**Figure 3D**). In comparison with the si-NC group, miR-1204 expression in Huh7 and SMMC-7721 cells with circ_0000098 knockdown was observably increased (**Figure 3E**). Additionally, miR-1204 expression in HCC tissues and cell lines, compared with that in adjacent tissues or HL-7702 cells, was markedly elevated (**Figures 3F, G**). In addition, miR-1204 expression was negatively correlated with circ_0000098 expression in HCC tissues (**Figure 3H**). Overall, these data indicated that circ_0000098 could directly bind to miR-1204 and restrain its expression.

**Table 3:** Correlation between miR-1204 expression and clinicopathological features in 34 HCC patients.

Characteristics	Number	miR-1204 expression		*P* value
		Low	High	
Age(years)				
≤50	13	6	7	1.000
>50	21	11	10	
Gender				
Male	25	11	14	0.437
Female	9	6	3	
TMN stage				
I/II	10	6	4	0.707
III/IV	24	11	13	
Tumor size (cm)				
<5	13	9	4	0.158
≥5	21	8	13	
Vascular invasion				
Yes	20	6	14	0.015*
No	14	11	3	
AFP (μg/L)				
≤400	12	6	6	0.720
>400	22	11	11	
Aetiology				
N.A.	9	6	3	0.292
Hepatitis B	15	8	7	
Alcoholic liver disease	6	1	5	
Fatty liver disease	4	2	2	

P value was calculated by Fisher’s exact test. *denotes p values less than 0.05.

### Circ_0000098 Could Inhibit HCC by Targeting miR-1204

**Figure 4:**
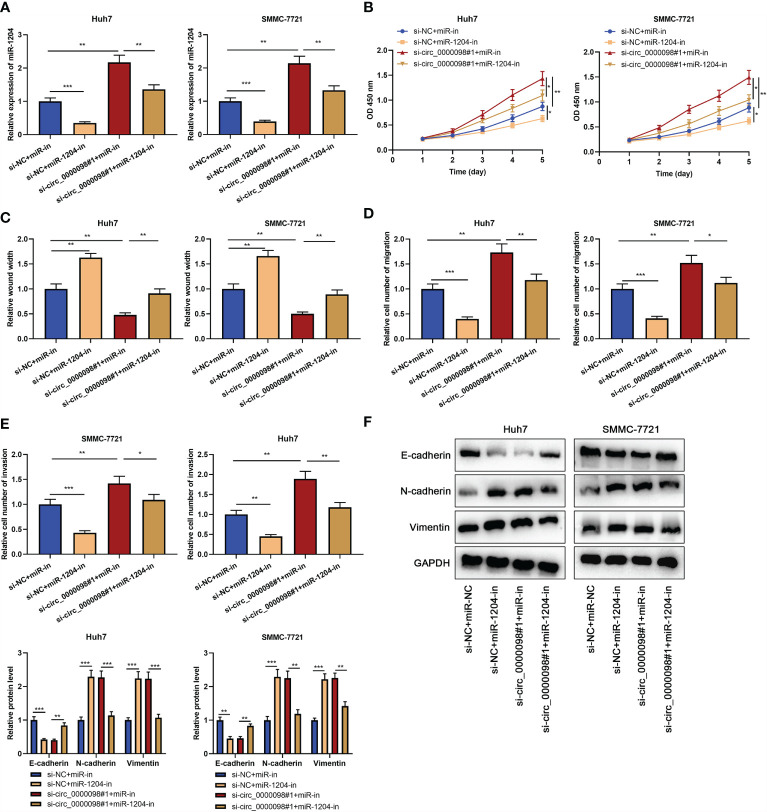
Circ_0000098 regulated the progression of HCC by adsorbing miR-1204. HCC cells were transfected with si-NC+miR-in, si-NC+miR-1204-in, si-circ_0000098#1+miR-in, or si-circ_0000098#1+miR-1204-in. **(A)** The expression of miR-1204 in Huh7 and SMMC-7721 cells was detected by qRT-PCR. **(B)** The cell proliferation was detected by CCK-8 assay. **(C)** The migration ability of cells was detected by wound healing assay. **(D, E)** Transwell assay was used to detect cell migration and invasion. **(F)** Western blot assay was used to detect the expression levels of E-cadherin, N-cadherin, and Vimentin in Huh7 and SMMC-7721 cells. *P < 0.05, **P < 0.01, and ***P < 0.001.

To study whether circ_0000098 partakes in regulating the progression of HCC through sponging miR-1204, we transfected si-NC+miR-in, si-NC+miR-1204-in, si-circ_0000098#1+miR-in, or si-circ_0000098#1+miR-1204-in into Huh7 and SMMC-7721 cells, respectively. qRT-PCR showed that miR-1204 expression was markedly declined in si-NC+miR-1204-in group as against si-NC+miR-in group, while miR-1204 expression level in si-circ_0000098#1+miR-in group was higher; miR-1204 expression in si-circ_0000098#1+miR-1204-in group, compared with that in si-circ_0000098#1+miR-in group, was decreased observably (**Figure 4A**). CCK-8, wound healing, Transwell, and Western blot assays found that in comparison with si-NC+miR-in group, the transfection of miR-1204 inhibitors markedly impeded the proliferation, migration, invasion, and EMT of Huh7 and SMMC-7721 cells; as against si-circ_0000098#1+miR-in group, the transfection of miR-1204 inhibitors weakened the promotion of circ_0000098 knockdown on proliferation, migration, invasion, and EMT of HCC cells (**Figures 4B**–**F**).

### Circ_0000098 Participated in the Progression of HCC *via* Modulating the miR-1204/ALX4 Axis

**Figure 5:**
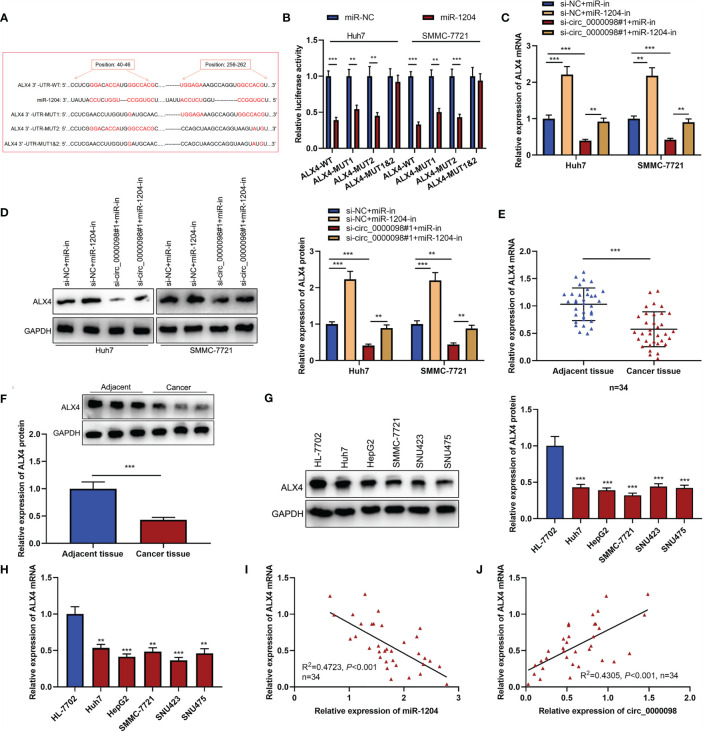
Circ_0000098 promoted the expression of ALX4 by sponging miR-1204. **(A)** The binding sequence between miR-1204 and ALX4 was predicted by TargetScan tool (http://www.targetscan.org/). **(B)** The binding relationship between miR-1204 and ALX4 was detected by dual-luciferase reporter assay. **(C, D)** qRT-PCR and Western blot were used to detect the effects of circ_0000098 and miR-1204 on ALX4 expression in Huh7 and SMMC-7721 cells. **(E-H)** qRT-PCR and Western blot were used to detect the expressions of ALX4 mRNA and protein in HCC tissues and cells. **(I, J)** Pearson’s correlation analysis was used to analyze the correlation between ALX4 mRNA and miR-1204 or circ_0000098 expressions in 34 HCC tissues. **P < 0.01 and ***P < 0.001.

To elaborate on the potential mechanism of circ_0000098/miR-1204 axis in HCC, we predicted the potential target gene of miR-1204 by the TargetScan tool (http://www.targetscan.org/) (17) and discovered that there were complementary binding sites between miR-1204 and ALX4 3’-UTR (**Figure 5A**). Notably, low expression of ALX4 expression was associated with the vascular invasion of the enrolled HCC patients (**Table 4**). Dual-luciferase reporter assay confirmed that miR-1204 overexpression markedly inhibited the luciferase activity of ALX4-WT reporter in Huh7 and SMMC-7721 cells, but the luciferase activity of ALX4-MUT reporter was not significantly changed (**Figure 5B**). Next, we studied the effects of the circ_0000098/miR-1204 axis on ALX4 expression and found that knocking down circ_0000098 inhibited ALX4 mRNA and protein expressions compared with the control while the transfection of miR-1204 inhibitors partially reversed these effects (**Figures 5C, D**). In addition, ALX4 mRNA and protein expressions were observably declined in HCC tissues and cells compared with that in adjacent tissues or HL-7702 cells (**Figures 5E**–**H**). Pearson’s analysis showed that ALX4 mRNA expression was negatively correlated with miR-1204 expression and positively with circ_0000098 expression in HCC tissues (**Figures 5I, J**). Next, we transfected miR-in, miR-1204-in, miR-1204-in+si-NC, and miR-1204-in+si-ALX4 into Huh7 and SMMC-7721 cells, and the successful transfection was confirmed by Western blot (**Supplementary Figure 2A**). We demonstrated that, the inhibitory effects of inhibiting miR-1204 on the proliferation, migration, invasion, and EMT of Huh7 and SMMC-7721 cells were reversed by ALX4 knockdown (**Supplementary Figures 2B**–**F**), and these data implied that miR-1204 promoted the malignant biological behaviors of HCC cells *via* repressing ALX4. Collectively, these results substantiated that ALX4 was a downstream target of miR-1204 in HCC cells, and its expression was negatively modulated by miR-1204 and positively by circ_0000098.

**Table 4:** Correlation between ALX4 expression and clinicopathological features in 34 HCC patients.

Characteristics	Number	ALX4 expression		*P* value
		Low	High	
Age(years)				
≤50	13	6	7	1.000
>50	21	11	10	
Gender				
Male	25	14	11	0.437
Female	9	3	6	
TMN stage				
I/II	10	4	6	0.707
III/IV	24	13	11	
Tumor size (cm)				
<5	13	5	8	0.480
≥5	21	12	9	
Vascular invasion				
Yes	20	14	6	0.015*
No	14	3	11	
AFP (μg/L)				
≤400	12	3	9	0.073
>400	22	14	8	
Aetiology				
N.A.	9	5	4	0.498
Hepatitis B	15	9	6	
Alcoholic liver disease	6	2	4	
Fatty liver disease	4	1	3	

P value was calculated by Fisher’s exact test. *denotes p values less than 0.05.

### Knockdown of Circ_0000098 Promoted the Lung Metastasis of HCC Cells *In Vivo*

**Figure 6:**
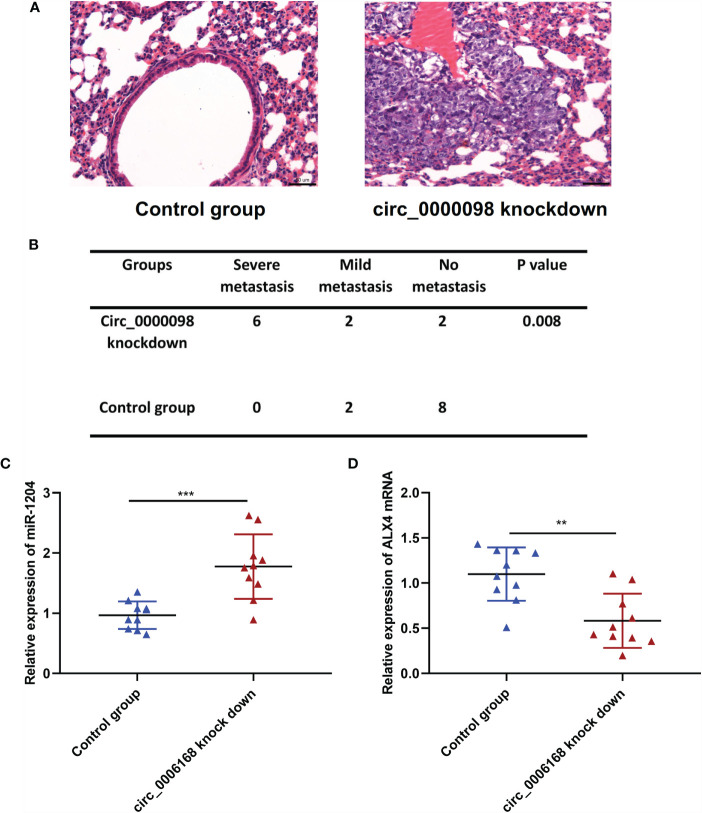
Circ_0000098 knockdown promotes the lung metastasis of Huh7 cells *in vivo*. **(A)** H&E staining was used to detect the metastatic nodule of the mice, which were injected with Hu7 cells with circ_0000098 knockdown or the control cells, and the representative images were shown. **(B)** The statistical analysis of the lung metastasis of the nude mice in the control group and circ_0000098 knockdown group. **(C, D)** qRT-PCR was used to detect the expression of miR-1204 and ALX4 mRNA in the lung tissues of the circ_0000098 knockdown group and the control group. **P < 0.01 and ***P < 0.001.

To further consolidate that circ_0000098 suppressed HCC progression, Huh7 cells with circ_0000098 knockdown and the control cells were respectively injected into the tail vein of the nude mice to establish a lung metastasis model. H&E staining of the lung tissues of the mice showed that, the lung metastasis of circ_0000098 knockdown group was much severer than that in the control group (**Figures 6A, B**). In addition, we found that compared with the control group, miR-1204 was highly expressed and ALX4 mRNA was lowly expressed in the lung tissues of mice in the circ_0000098 knockdown group (**Figures 6C, D**). In summary, these data indicate that circ_0000098 may inhibit the progression of HCC by suppressing metastasis.

## Discussion

Unlike other non-coding RNA, such as miRNA and long non-coding RNA, circRNA is highly stable (18). CircRNAs play a key role in the pathogenesis of human diseases, including cancer (19, 20). It is reported that the abnormal expression of circRNA is associated with the turmorigenesis and progression of HCC. For instance, circ-IGF1R expression level is raised in HCC, which can promote proliferation and reduce the apoptosis in HCC by activating PI3K/AKT pathway (21); circ_0003418, a tumor-suppressive circRNA in HCC, can enhance the sensitivity of HCC cells to cisplatin by inhibiting Wnt/β-catenin pathway (22); circ-ADD3 promotes EZH2 degradation through CDK1-mediated ubiquitination, thus inhibiting the metastasis of HCC cells (23). Here, we proved that circ_0000098 was observably lowly expressed in HCC tissues and cells, which was related to the tumor size of HCC. Functionally, we confirmed that knocking down circ_0000098 promoted the viability, migration, invasion, and EMT of HCC cells, while overexpression of circ_0000098 worked oppositely. These findings revealed the important role of circ_0000098 in HCC for the first time.

MiRNA, non-coding RNA with 19-25 *nt*, can negatively modulate gene expression *via* binding to the 3’UTR of target mRNAs (24). As a vital factor in regulating cell proliferation, differentiation, and apoptosis, miRNA, as reported, modulates the progression of various tumors, including HCC (13). For example, miR-330-5p accelerates the progression of HCC by targeting SPRY2 to activate MAPK/ERK signaling (25); miR-185-5p targets ROCK2 and restrains migration and invasion of HCC cells (26). In addition, circRNA can exert its effect *via* many mechanisms, among which the ceRNA mechanism is the most widely investigated (27). For example, circSETD3 inhibits the growth of HCC cells by adsorbing miR-421 (28); circ-FOXP1 accelerates the progression of HCC through adsorbing miR-875-3p and miR-421 (29). In this study, we found that circ_0000098 could interact with miR-1204 in HCC cells. In addition, miR-1204 expression level was raised in HCC tissues and cell lines, which was consistent with the previous report (13). We also demonstrated that transfection of miR-1204 inhibitors could remarkably inhibit the effects of circ_0000098 knockdown on the malignant biological behaviors of HCC cells, highlighting that circ_0000098 could regulate HCC progression through sponging miR-1204.

ALX4 is a member of the PRD subfamily of the homeobox gene (30). ALX4 is widely expressed in various tissues and organs, and features prominently in embryonic development and epidermal growth (31, 32). Previous studies authenticate that the hypermethylation of ALX4 gene is found in colorectal cancer and bladder cancer tissues, which can be used as a candidate biomarker (33, 34); ALX4 expression is decreased in breast cancer tissues and cells, and it inhibits the progression of breast cancer by interfering with Wnt/β-catenin pathway (35); in ovarian cancer, ALX4 promotes invasion and EMT of ovarian cancer cells by raising SLUG expression (36); ALX4 expression is declined in HCC tissues, and ALX4 overexpression impedes the multiplication, invasion, and EMT process of HCC cells (37). Here, we also found that ALX4 expression was down-regulated in HCC tissues and cells. Besides, ALX4 was identified as the direct target of miR-1204 in HCC cells. Additionally, our data supported that circ_0000098 positively regulated ALX4 expression by competitively binding with miR-1204. In addition, we found that ALX4 knockdown reversed the inhibitory effects of miR-1204 down-regulation on HCC cell proliferation, migration, invasion, and EMT. Therefore, it was concluded that circ_0000098/miR-1204/ALX4 regulatory network was involved in the progression of HCC.

There are some limitations of the present work. Firstly, even though it was revealed that circ_0000098 was down-regulated in HCC tissues, the mechanism of its dysregulation is still unclear. Additionally, the sample number of the present work is relatively small, and in the following work, a larger cohort with the patients from different medical centers will help evaluate the significance of circ_0000098, miR-1204 and ALX4 as biomarkers. Notably, a recent study reports that up-regulated serum miR-1204 level implies worse prognosis of breast cancer patients (38). Whether the serum miR-1204 level is associated with the prognosis of HCC patients remains to be explored in the future.

To recapitulate briefly, circ_0000098 is lowly expressed in HCC tissues and cells, and its low expression level is associated with larger tumor size and advanced TNM stage of HCC. Besides, circ_0000098 can restrain the multiplication, migration, invasion, and EMT of HCC cells *via* targeting miR-1204 to up-regulate ALX4 expression. Our study provides novel explanation to the molecular mechanism of HCC progression.

## Data Availability Statement

The original contributions presented in the study are included in the article/**Supplementary Material**. Further inquiries can be directed to the corresponding authors.

## Ethics Statement

Our study was approved by the Ethics Review Board of Yantai Affiliated Hospital of Binzhou Medical University. The procedures of clinical specimen collection were in compliance with the Declaration of Helsinki. The patients/participants provided their written informed consent to participate in this study. The *in vivo* study was approved by the Animal Care and Use Committee of Yantai Affiliated Hospital of Binzhou Medical University.

## Author Contributions

WJY and ML designed the study and experiments. ML, QL, and WYY collected clinical samples and performed the experiments. XLC conducted the data analysis. ML, WJY, and QL drafted the manuscript. ML and WJY reviewed and revised the manuscript. All authors contributed to the article and approved the submitted version.

## Funding

The study was funded by Yantai Science and Technology Project (2016WS062). Shandong Province Medicine and Health Science and Technology Plan Development Project (NO: 202003031068).

## Conflict of Interest

The authors declare that the research was conducted in the absence of any commercial or financial relationships that could be construed as a potential conflict of interest.

## Publisher’s Note

All claims expressed in this article are solely those of the authors and do not necessarily represent those of their affiliated organizations, or those of the publisher, the editors and the reviewers. Any product that may be evaluated in this article, or claim that may be made by its manufacturer, is not guaranteed or endorsed by the publisher.

## Supplementary Material

Supplementary Figure 1 Overexpression of circ_0000098 inhibited proliferation, migration, invasion, and EMT of HCC cells. **(A)** qRT-PCR was used to detect the expression of circ_0000098 in Huh7 and SMMC-7721 cells after the transfection of circ_0000098 overexpression plasmid. **(B)** CCK-8 assay was used to detect cell proliferation after overexpression of circ_0000098. **(C)** Wound healing assay was used to detect the migration ability of cells after overexpression of circ_0000098 (Scale bar, 100 μm). **(D, E)** Transwell assay was used to detect cell migration and invasion after overexpression of circ_0000098 (Scale bar, 250 μm). **(F)** Western blot assay was used to detect the expression levels of E-cadherin, N-cadherin, and Vimentin in Huh7 and SMMC-7721 cells overexpressing circ_0000098. ***P* < 0.01 and ****P* < 0.001.

Supplementary Figure 2 MiR-1204 regulated HCC cells proliferation, migration, invasion, and EMT *via* ALX4. HCC cells were transfected with miR-in, miR-1204-in, miR-1204-in+si-NC, and miR-1204-in+si-ALX4. **(A)** Western blot assay was used to detect the protein level of ALX4 in Huh7 and SMMC-7721 cells transfected with miR-1204 inhibitor or co-transfected with si-ALX4. **(B)** CCK-8 assay was used to detect the proliferation of Huh7 and SMMC-7721 cells after the transfection with miR-1204 inhibitor or co-transfection with si-ALX4. **(C–E)** Wound healing assay (Scale bar, 100 μm) and Transwell assay (Scale bar, 250 μm) were used to detect the migration and invasion of Huh7 and SMMC-7721 cells after the transfection with miR-1204 inhibitor or co-transfection with si-ALX4. **(F)** Western blot assay was used to detect the expression levels of E-cadherin, N-cadherin, and Vimentin in Huh7 and SMMC-7721 cells transfected with miR-1204 inhibitor or co-transfected with si-ALX4. **P* < 0.05, ***P* < 0.01, and ****P* < 0.001.
