# Supplementary figures and images for: Circular RNA Circ_0000098 Elevates ALX4 Expression via Adsorbing miR-1204 to Inhibit the Progression of Hepatocellular Carcinoma
Source: Front Oncol. 2021 Nov 26;11:696078. doi: 10.3389/fonc.2021.696078 (PMC8662564; doi:10.3389/fonc.2021.696078)

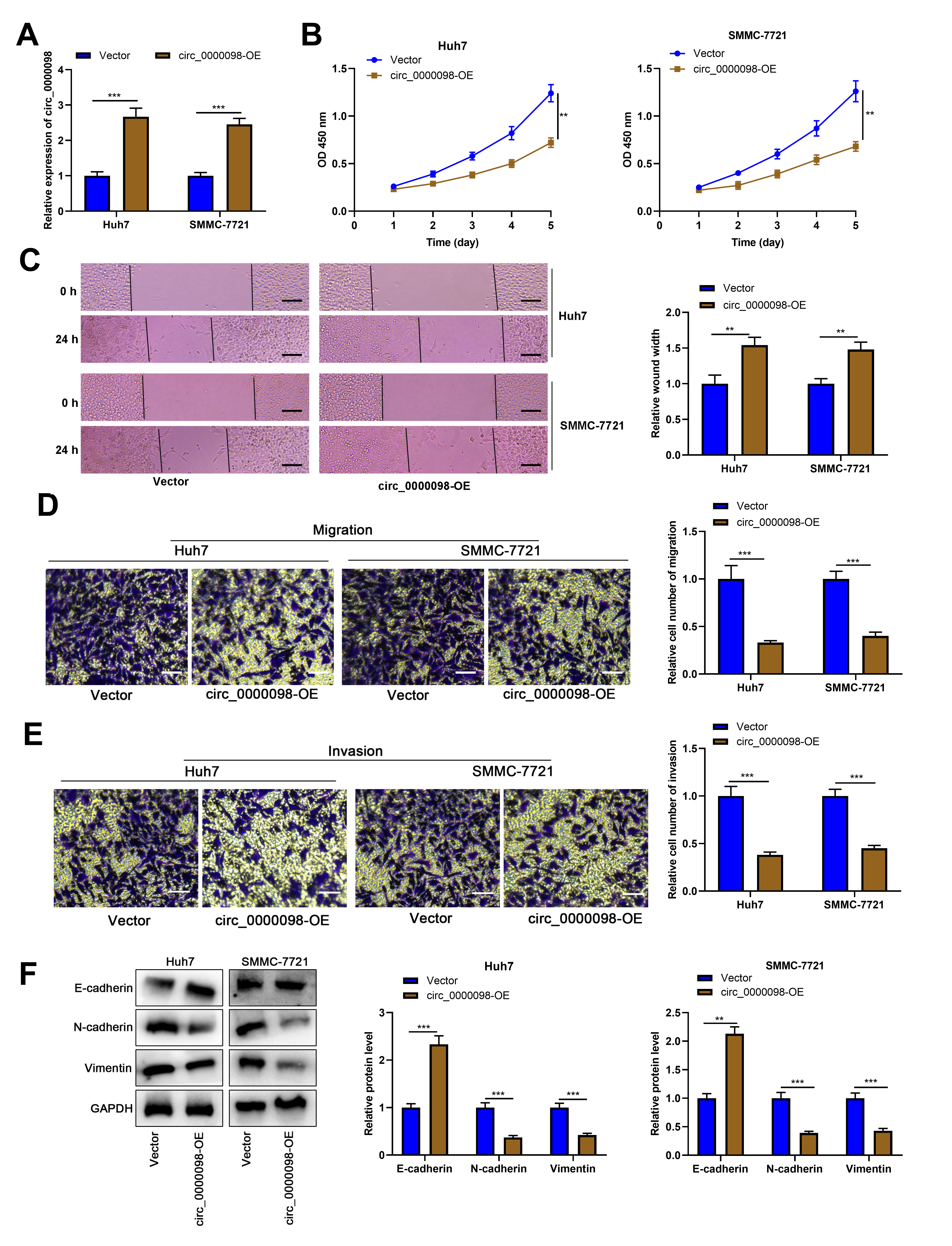

Supplement: Supplementary Figure 1 — Overexpression of circ_0000098 inhibited proliferation, migration, invasion, and EMT of HCC cells. (A) qRT-PCR was used to detect the expression of circ_0000098 in Huh7 and SMMC-7721 cells after the transfection of circ_0000098 overexpression plasmid. (B) CCK-8 assay was used to detect cell proliferation after overexpression of circ_0000098. (C) Wound healing assay was used to detect the migration ability of cells after overexpression of circ_0000098 (Scale bar, 100 μm). (D, E) Transwell assay was used to detect cell migration and invasion after overexpression of circ_0000098 (Scale bar, 250 μm). (F) Western blot assay was used to detect the expression levels of E-cadherin, N-cadherin, and Vimentin in Huh7 and SMMC-7721 cells overexpressing circ_0000098. **P < 0.01 and ***P < 0.001. [file Image_1.tif]

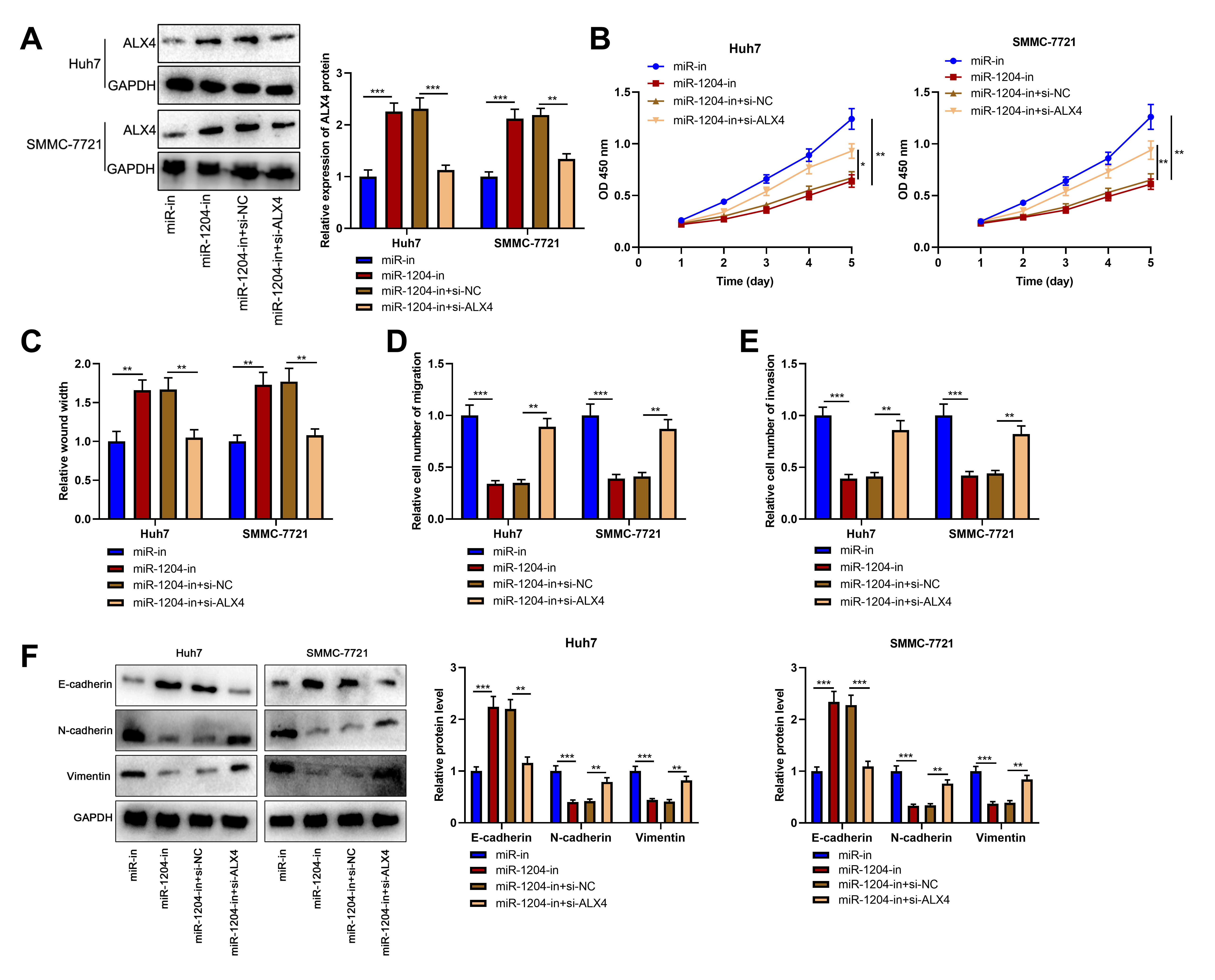

Supplement: Supplementary Figure 2 — MiR-1204 regulated HCC cells proliferation, migration, invasion, and EMT via ALX4. HCC cells were transfected with miR-in, miR-1204-in, miR-1204-in+si-NC, and miR-1204-in+si-ALX4. (A) Western blot assay was used to detect the protein level of ALX4 in Huh7 and SMMC-7721 cells transfected with miR-1204 inhibitor or co-transfected with si-ALX4. (B) CCK-8 assay was used to detect the proliferation of Huh7 and SMMC-7721 cells after the transfection with miR-1204 inhibitor or co-transfection with si-ALX4. (C–E) Wound healing assay (Scale bar, 100 μm) and Transwell assay (Scale bar, 250 μm) were used to detect the migration and invasion of Huh7 and SMMC-7721 cells after the transfection with miR-1204 inhibitor or co-transfection with si-ALX4. (F) Western blot assay was used to detect the expression levels of E-cadherin, N-cadherin, and Vimentin in Huh7 and SMMC-7721 cells transfected with miR-1204 inhibitor or co-transfected with si-ALX4. *P < 0.05, **P < 0.01, and ***P < 0.001. [file Image_2.tif]
