# Supplementary material for: Circular RNA Circ_0000098 Elevates ALX4 Expression via Adsorbing miR-1204 to Inhibit the Progression of Hepatocellular Carcinoma
Source: Front Oncol. 2021 Nov 26;11:696078. doi: 10.3389/fonc.2021.696078 (PMC8662564; doi:10.3389/fonc.2021.696078)

**The original image of Western blot experiment**

Supplementary Figure 1 Western blot assay was used to detect the expression levels of E-cadherin, N-cadherin, and Vimentin in Huh7 and SMMC-7721 cells with down-regulated circ_0000098 expression.

Huh7 cell

E-cadherin N-cadhenrin


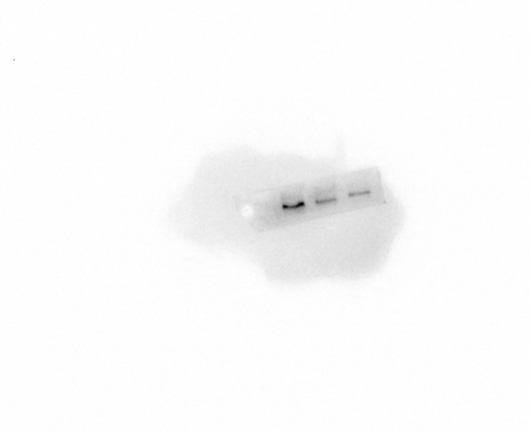

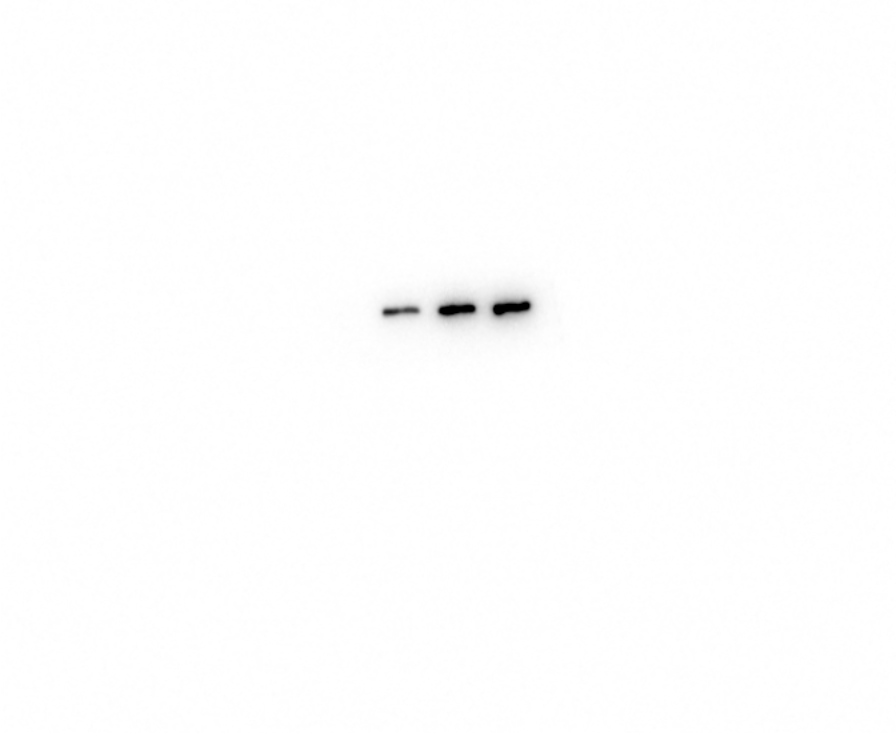


Vimentin GAPDH


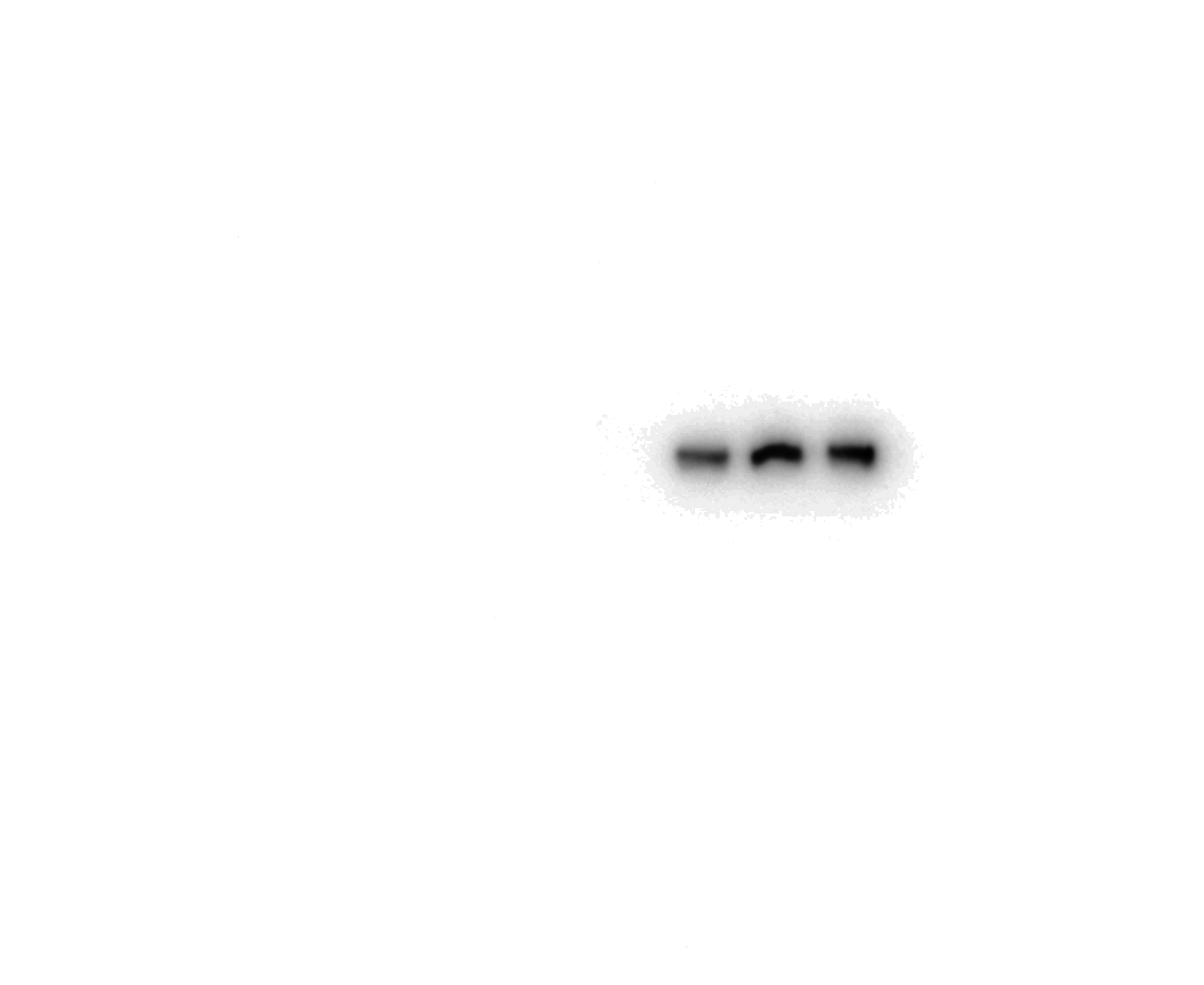

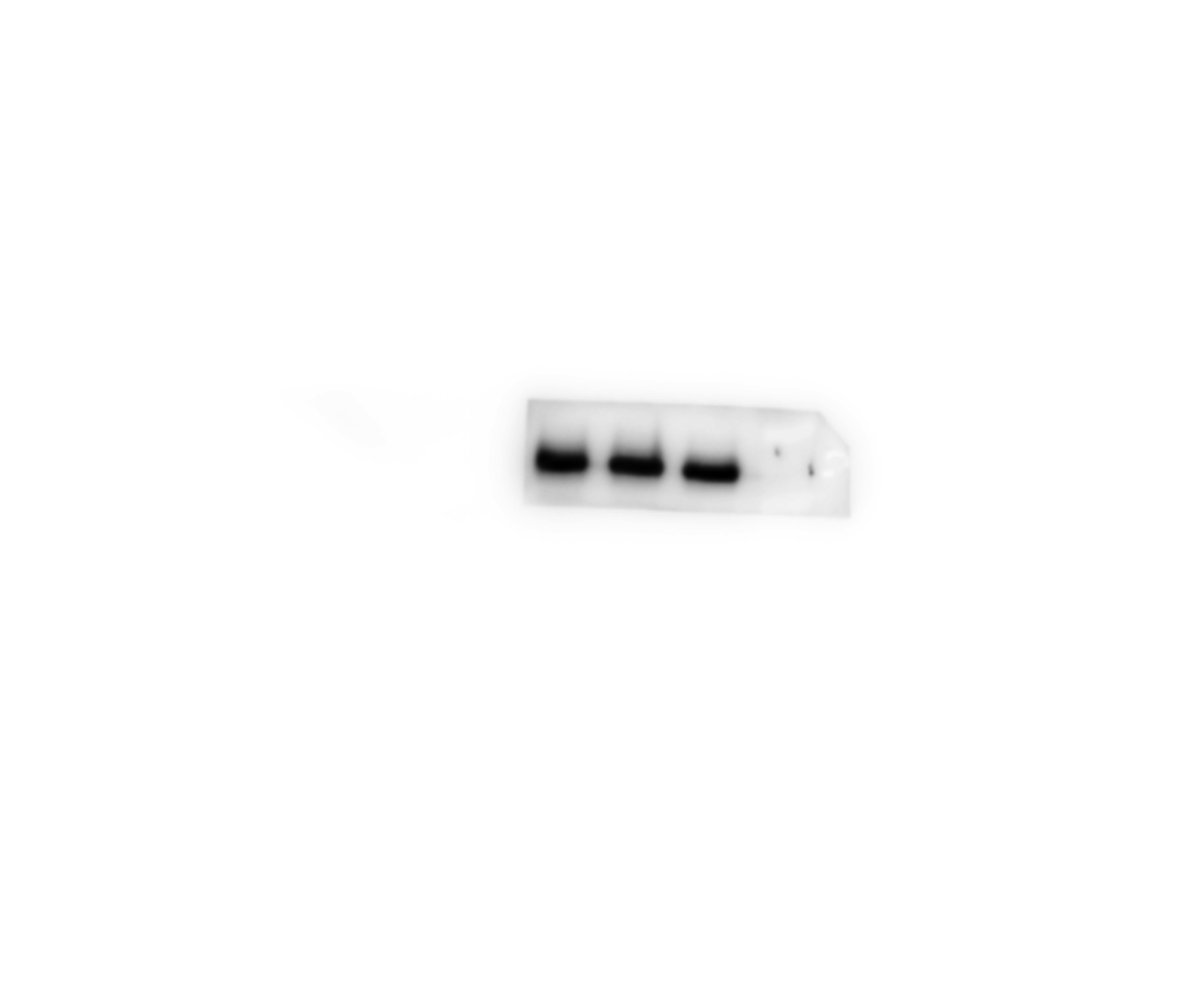


SMMC-7721 cell

E-cadherin N-cadhenrin


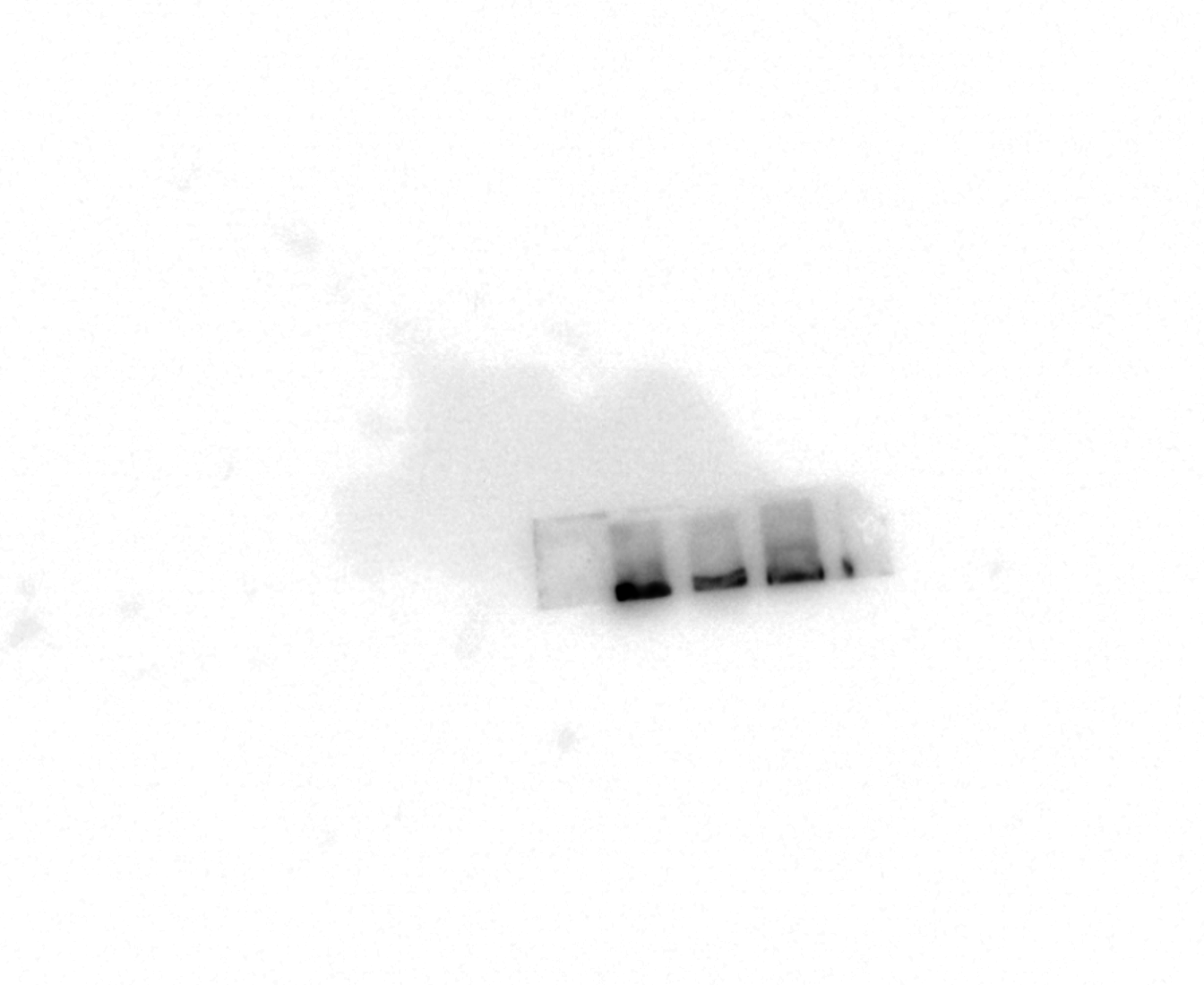

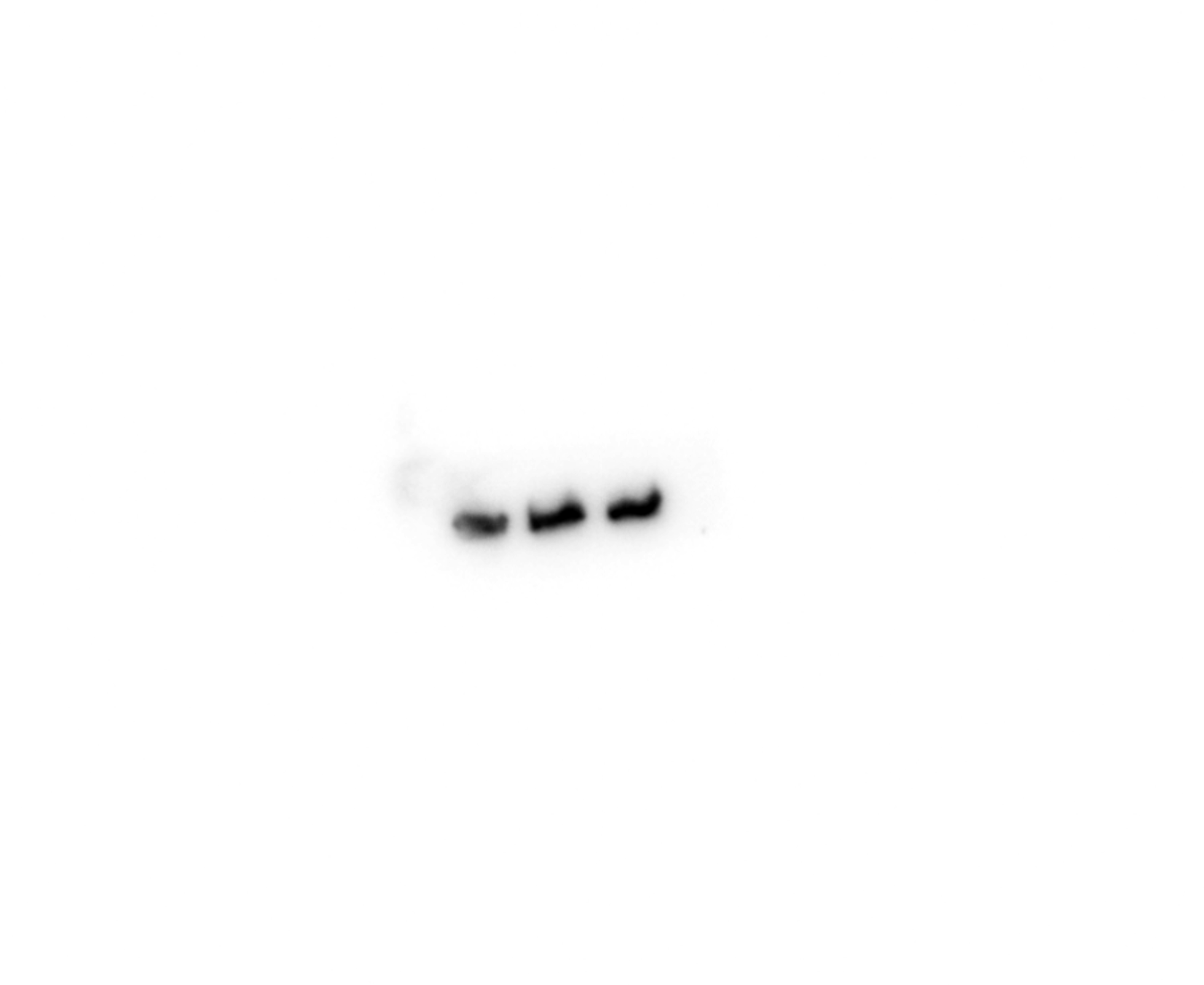


Vimentin GAPDH


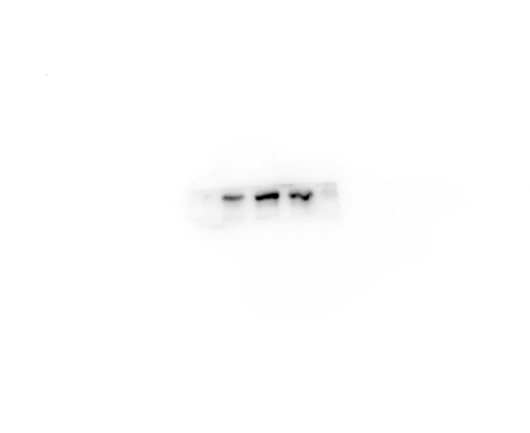

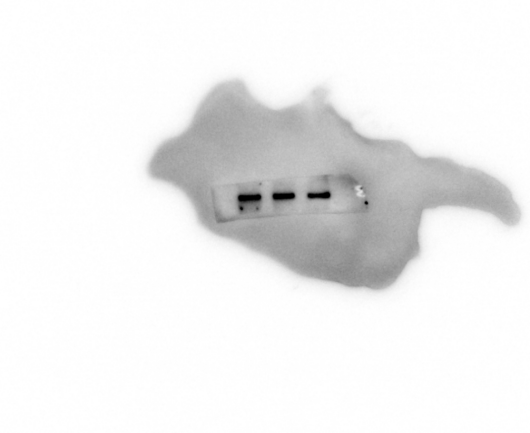


Supplementary Figure 2 Western blot assay was used to detect the expression levels of E-cadherin, N-cadherin, and Vimentin in Huh7 and SMMC-7721 cells.

Huh7 cell

E-cadherin N-cadhenrin


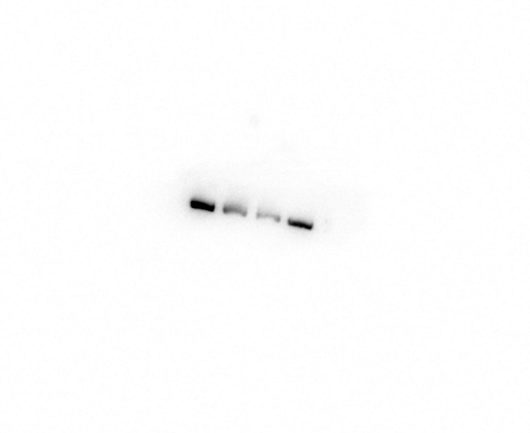

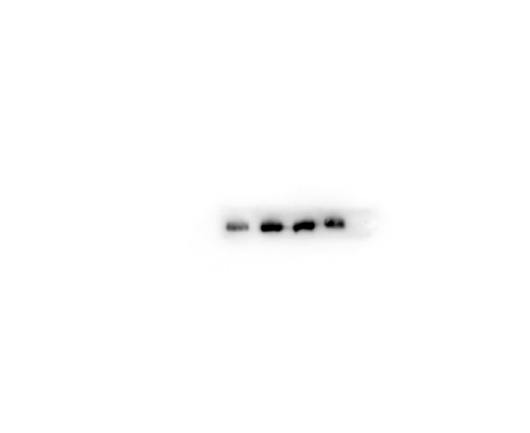


Vimentin GAPDH


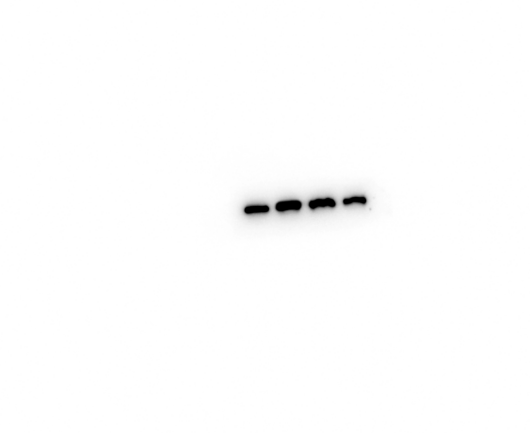

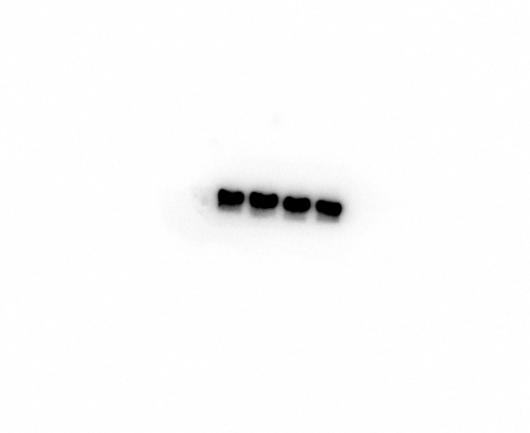


SMMC-7721 cell

E-cadherin N-cadhenrin


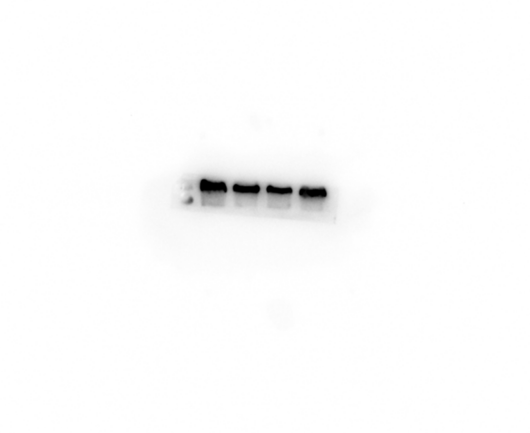

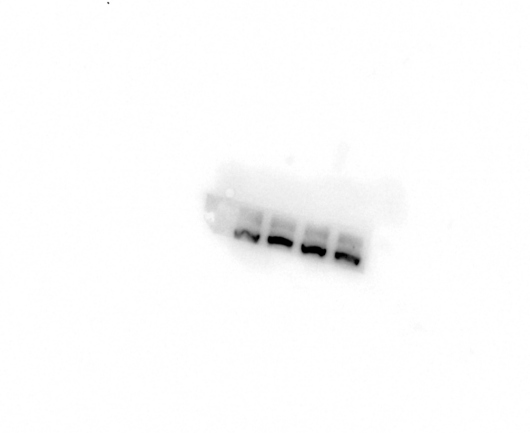


Vimentin GAPDH


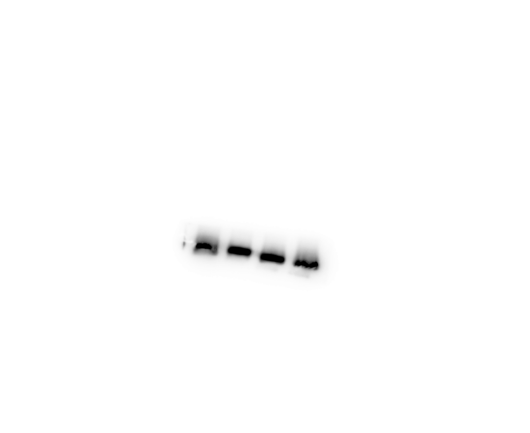

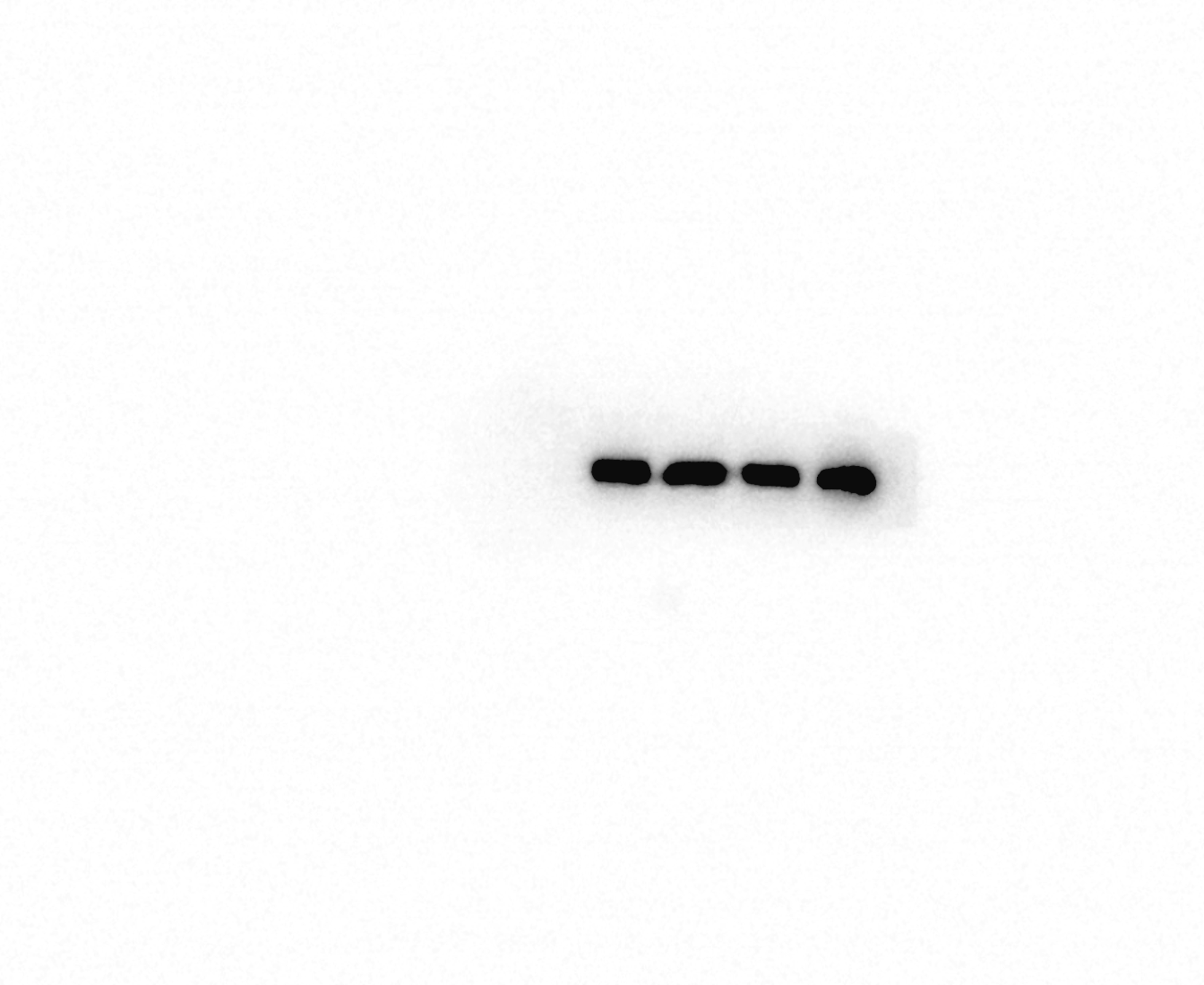


Supplementary Figure 3 Western blot was used to detect the effects of circ_0000098 and miR-1204 on ALX4 expression in Huh7 and SMMC-7721 cells.

Huh7 cell

ALX4 GAPDH


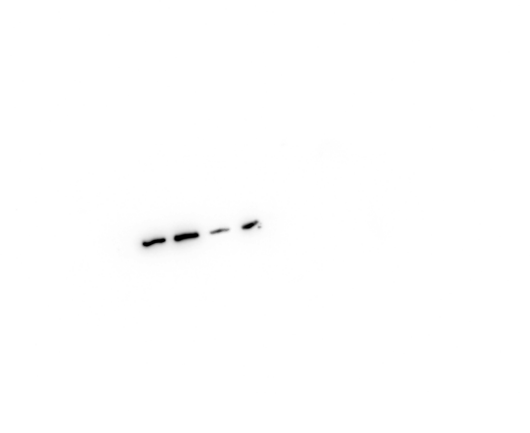

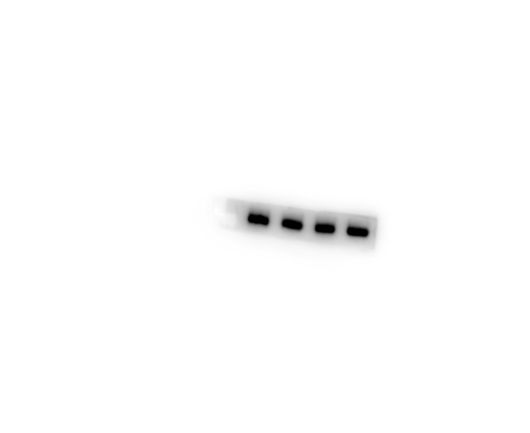


SMMC-7721 cell

ALX4 GAPDH


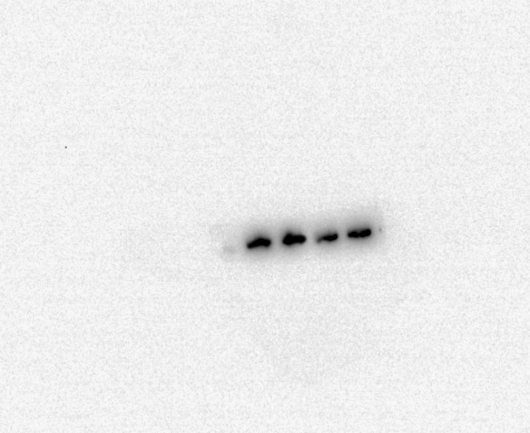

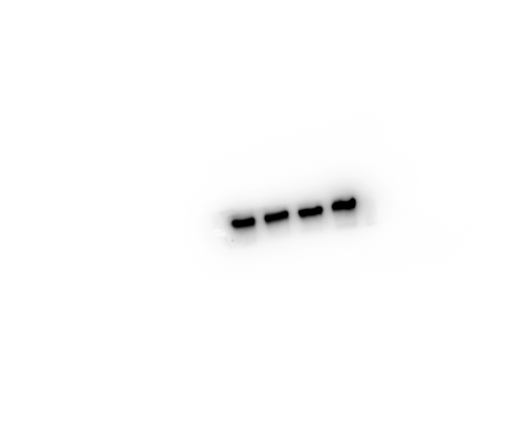


Supplementary Figure 4 Western blot were used to detect the expressions of ALX4 protein in HCC tissues and adjacent tissues.

ALX4 GAPDH


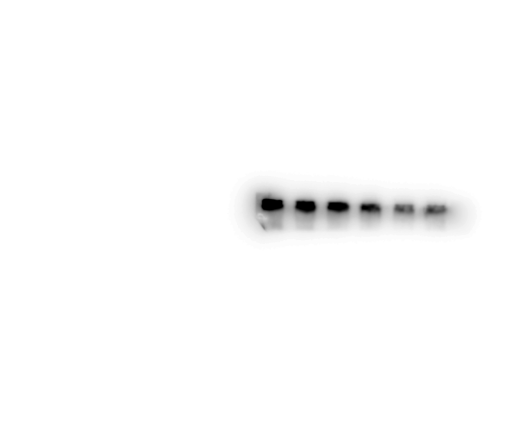

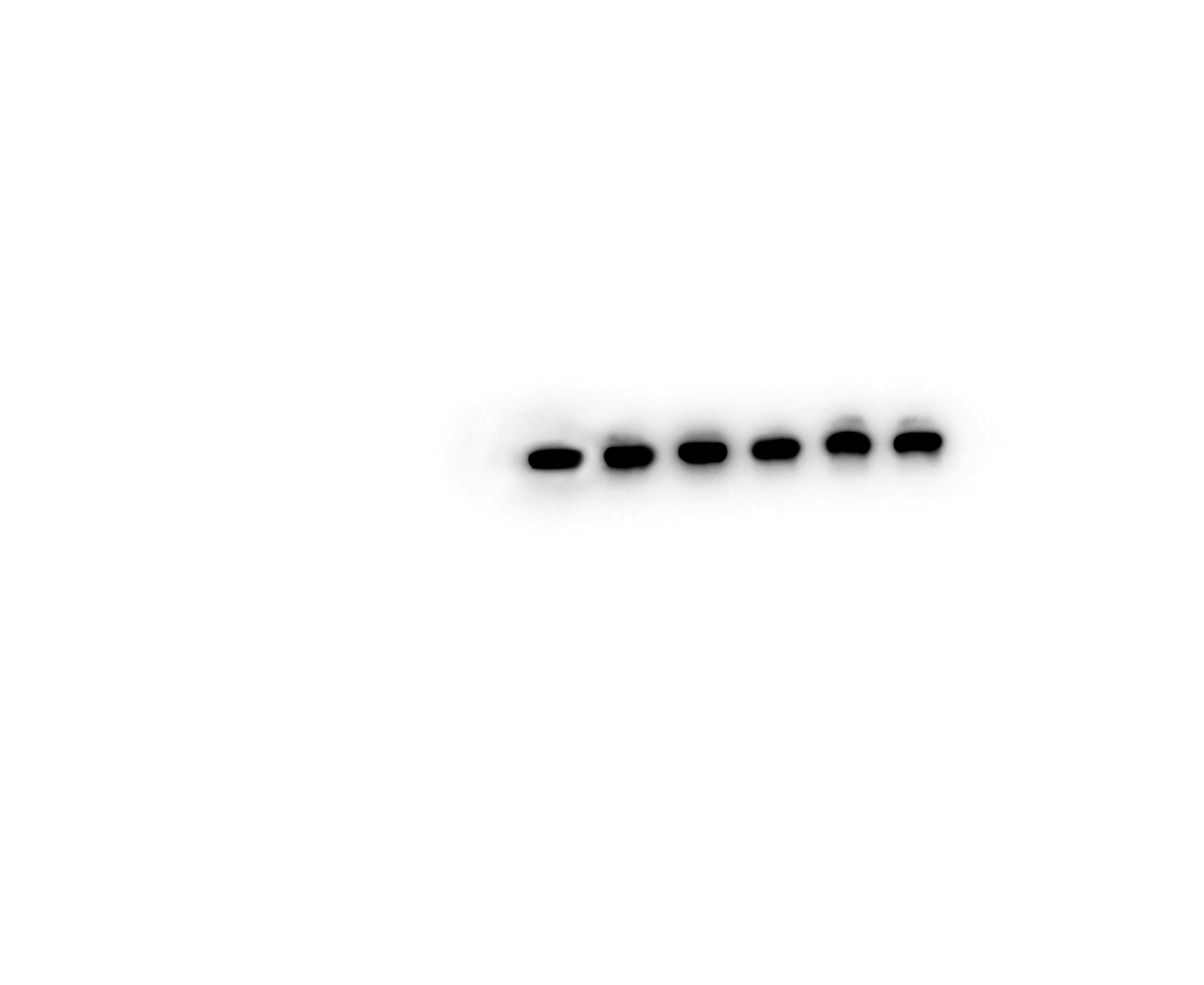


Supplementary Figure 5 Western blot were used to detect the expressions of ALX4 protein in HCC cells and HL-7702 cells.

ALX4 GAPDH


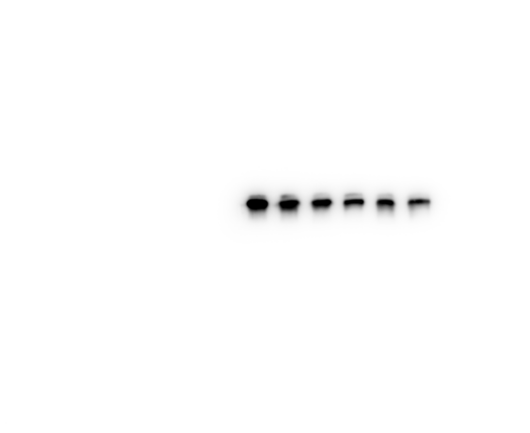

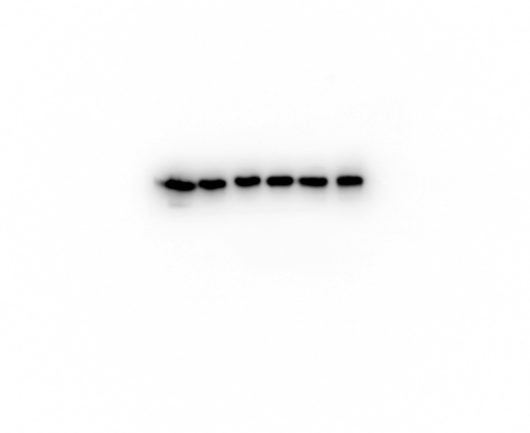


Supplementary Figure 6 Western blot assay was used to detect the expression levels of E-cadherin, N-cadherin, and Vimentin in Huh7 and SMMC-7721 cells overexpressing circ_0000098.

Huh7 cell

E-cadherin N-cadhenrin


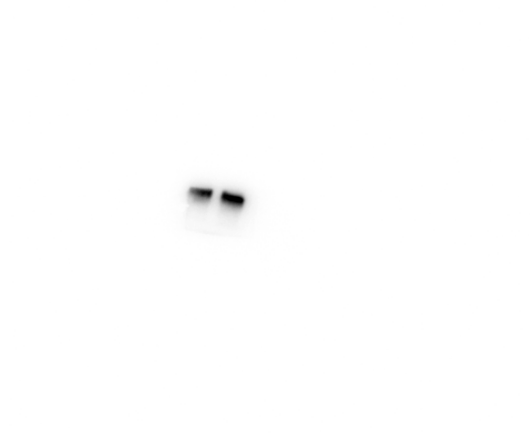

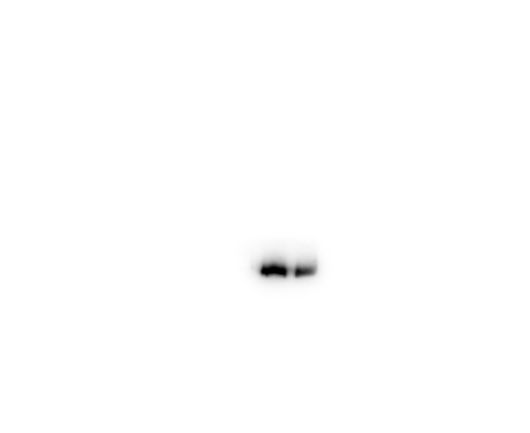


Vimentin GAPDH


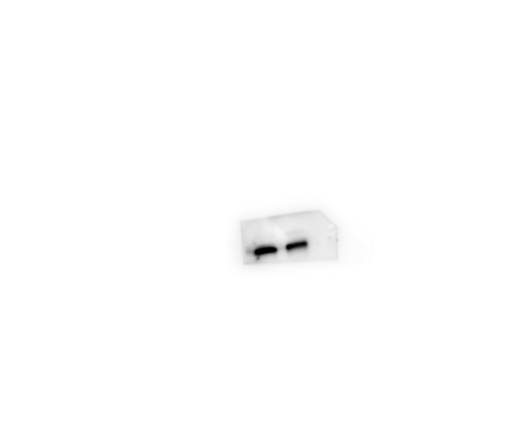

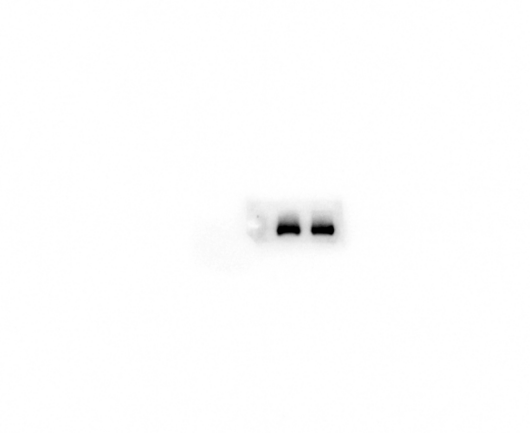


SMMC-7721 cell

E-cadherin N-cadhenrin


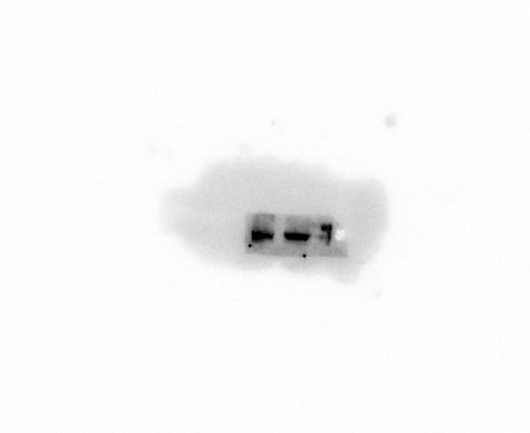

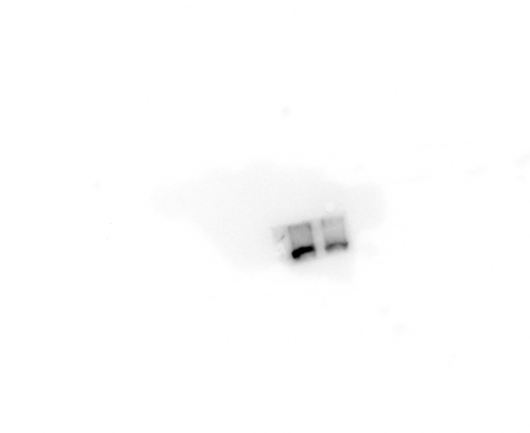


Vimentin GAPDH


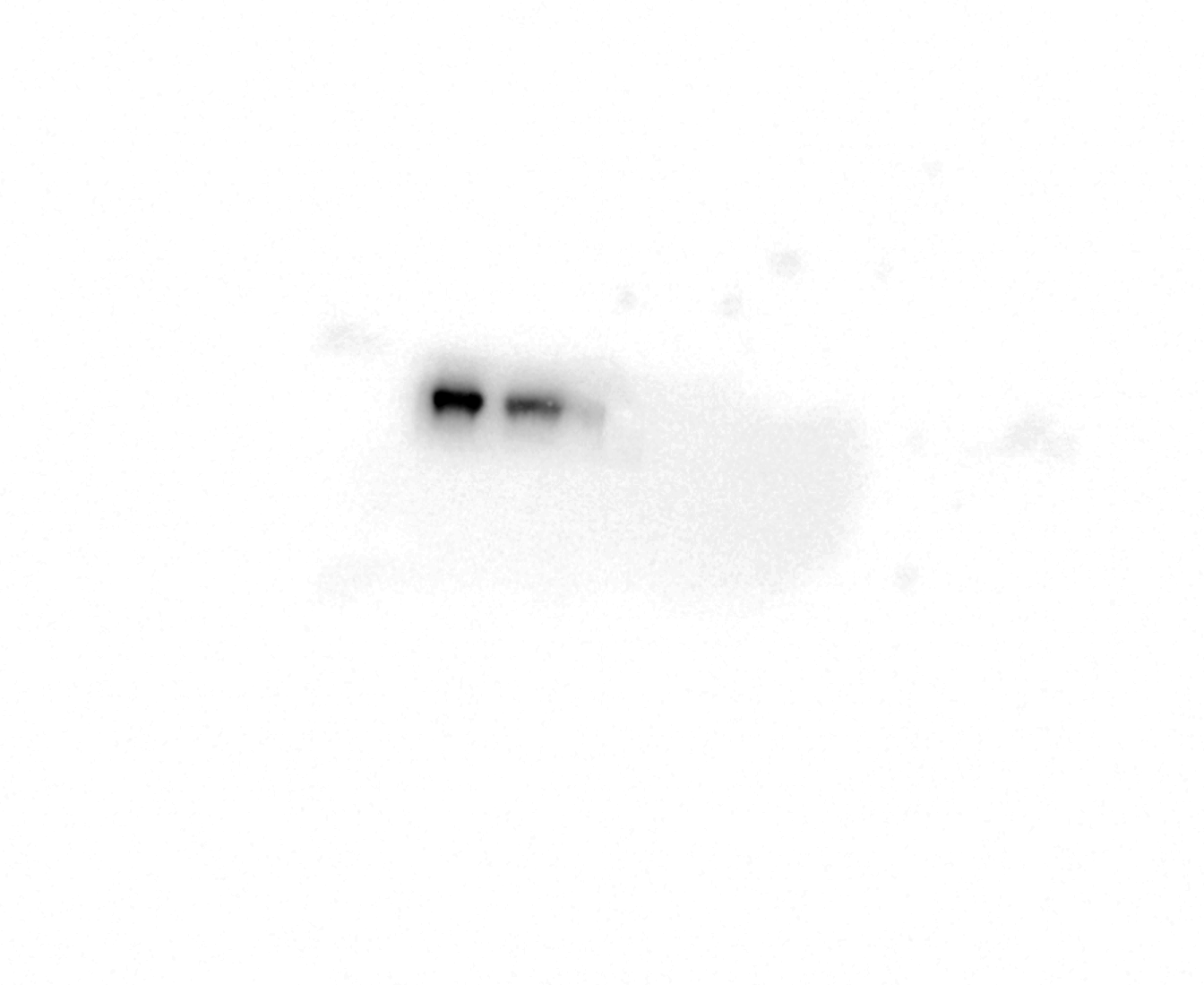

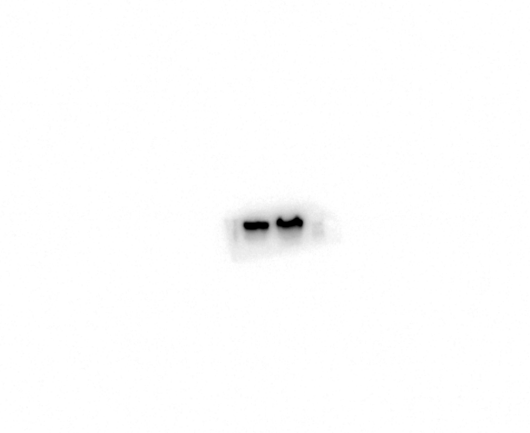


Supplementary Figure 7 Western blot assay was used to detect the protein level of ALX4 in Huh7 and SMMC-7721 cells transfected with miR-1204 inhibitor or co-transfected with si-ALX4.

Huh7 cell

ALX4 GAPDH


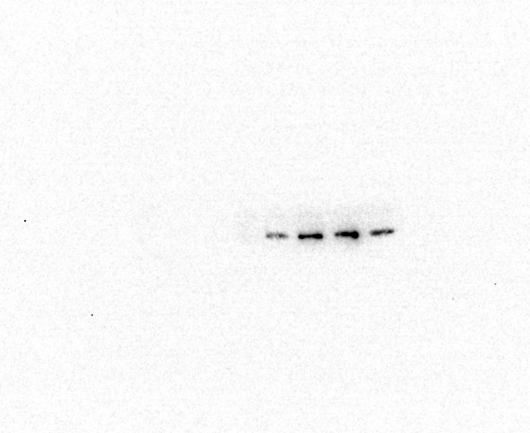

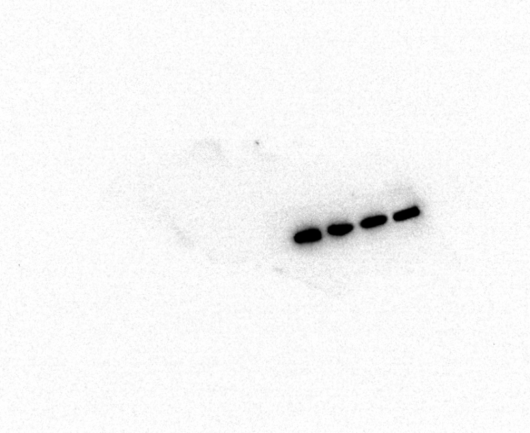


SMMC-7721 cell

ALX4 GAPDH


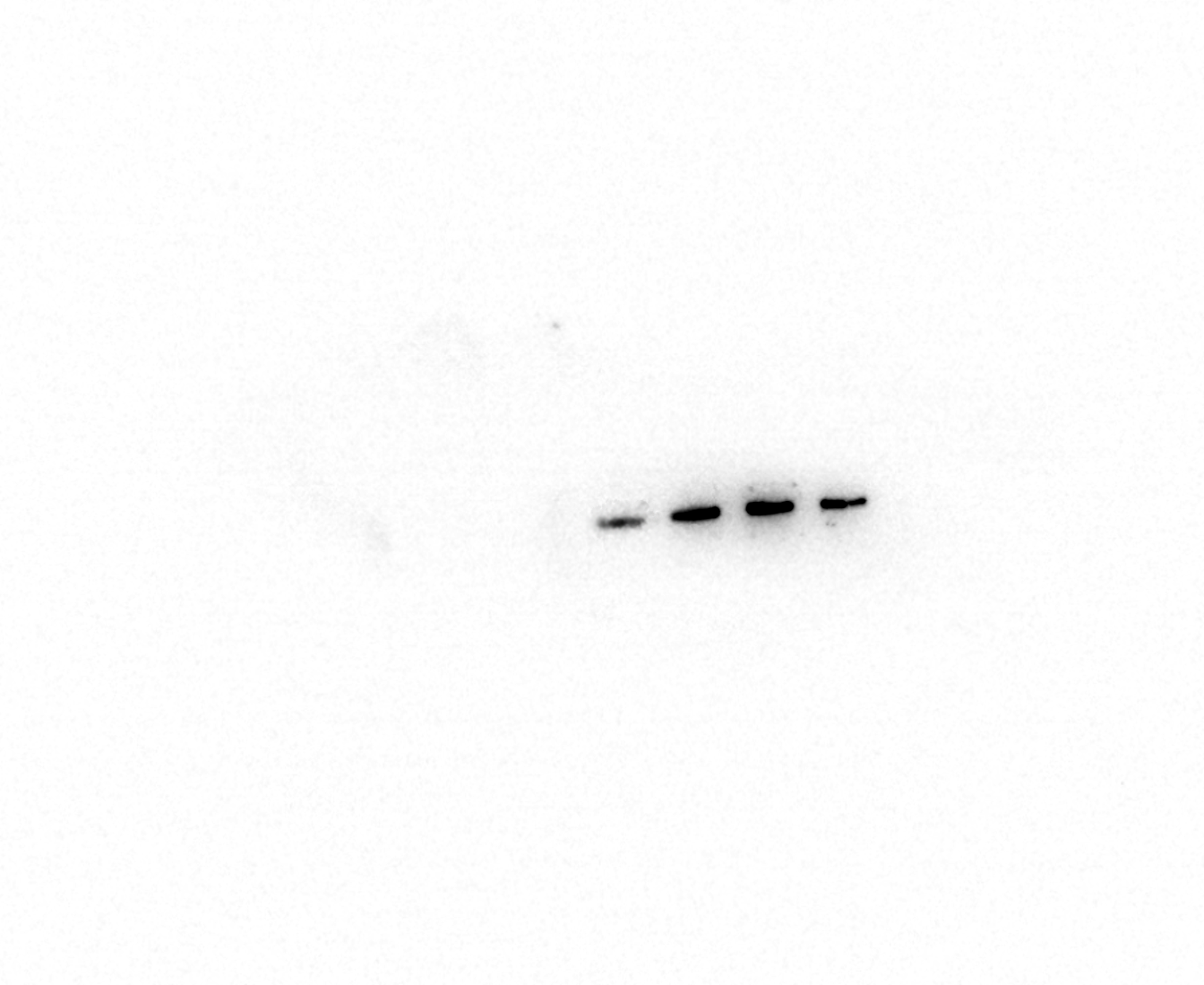

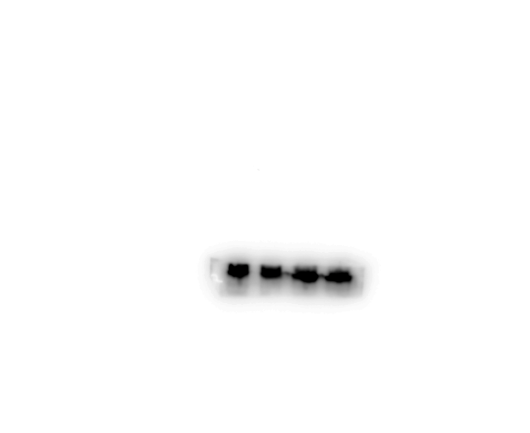


Supplementary Figure 8 Western blot assay was used to detect the expression levels of E-cadherin, N-cadherin, and Vimentin in Huh7 and SMMC-7721 cells transfected with miR-1204 inhibitor or co-transfected with si-ALX4.

Huh7 cell

E-cadherin N-cadhenrin


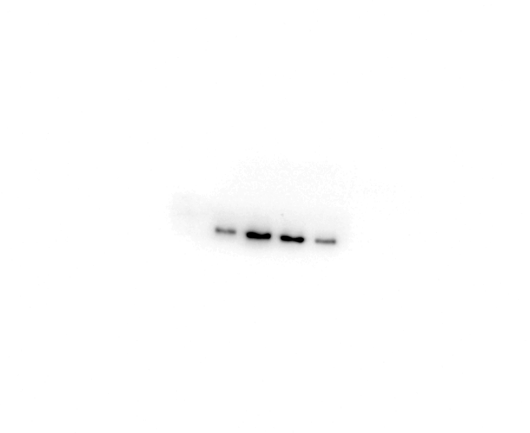

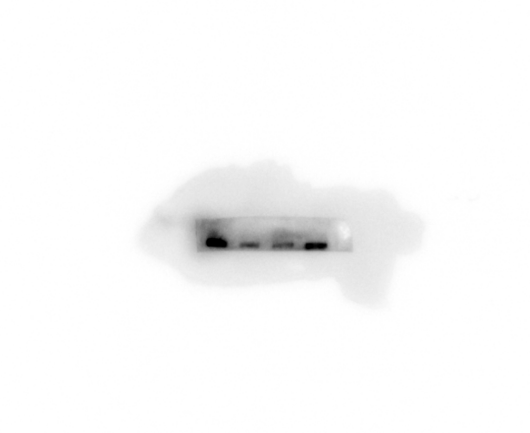


Vimentin GAPDH


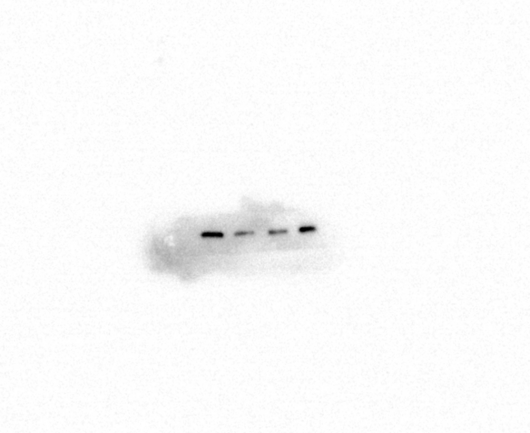

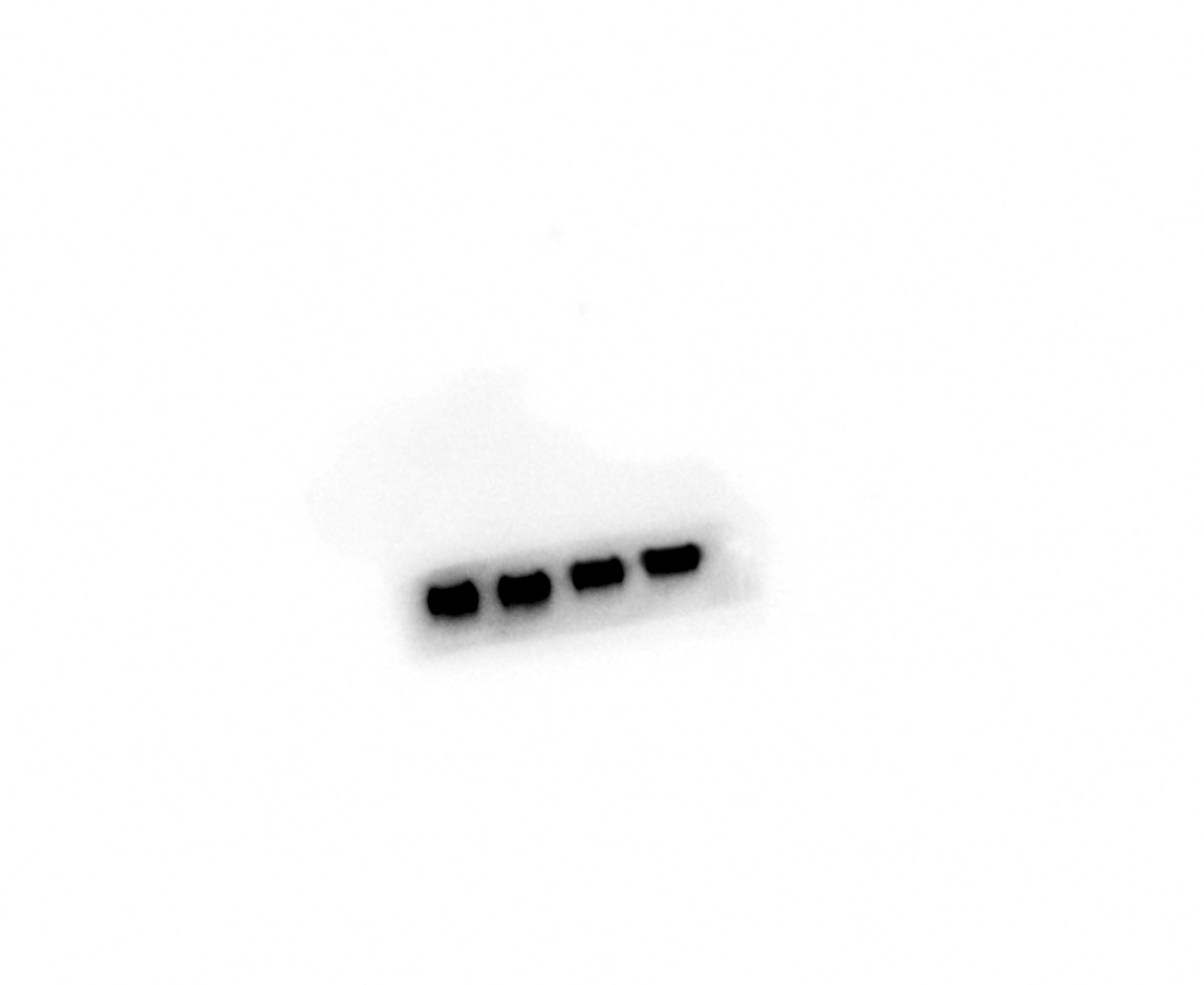


SMMC-7721 cell

E-cadherin N-cadhenrin


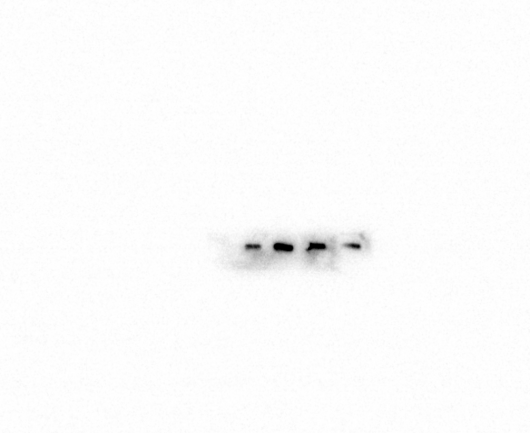

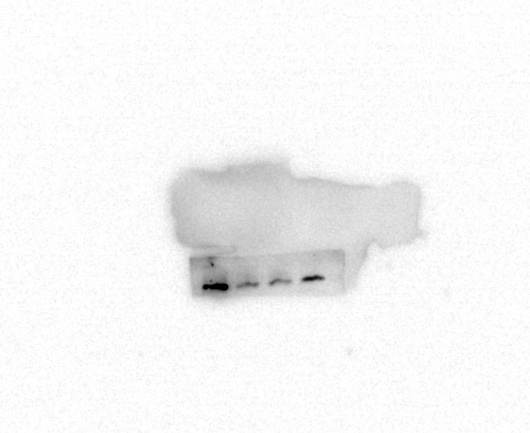


Vimentin GAPDH


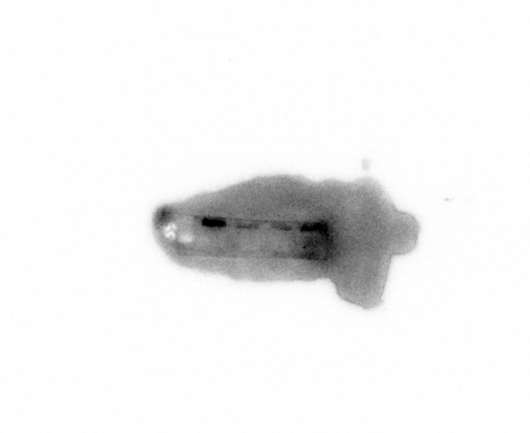

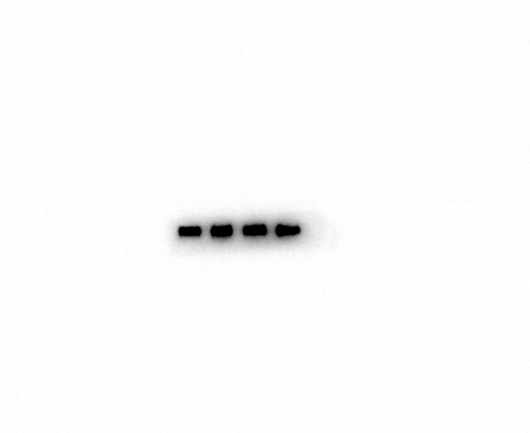

Supplement: Supplementary file 3 [file DataSheet_1.docx]
